# Probability of sequelae following *Campylobacter* spp. infections: Update of systematic reviews and meta‐analyses

**DOI:** 10.1002/puh2.145

**Published:** 2023-12-20

**Authors:** Elisabeth Schorling, Sebastian Knorr, Sonja Lick, Pablo Steinberg, Dagmar Adeline Brüggemann

**Affiliations:** ^1^ Department of Safety and Quality of Meat Max Rubner‐Institut, Federal Research Institute of Nutrition and Food Kulmbach Bavaria Germany; ^2^ Max Rubner‐Institut, Federal Research Institute of Nutrition and Food Karlsruhe Baden‐Wurttemberg Germany

**Keywords:** arthritis, reactive, *Campylobacter* infections, Guillain–Barré syndrome, inflammatory bowel diseases, irritable bowel syndrome, meta‐analysis

## Abstract

**Introduction:**

Reactive arthritis (REA) and Guillain–Barré syndrome (GBS) are postinfectious complications of *Campylobacter* enteritis (CE); associations with inflammatory bowel diseases and irritable bowel syndrome (IBS) are also discussed. The objective of this study was to summarize existing evidence on the probability of sequelae following confirmed CE.

**Methods:**

All studies included in previous reviews and meta‐analyses on this topic were retrieved and assessed for eligibility; a systematic literature search was conducted to collect more recent reports. For each sequela, random effects meta‐analyses were performed; the risk of bias and the quality of evidence were evaluated.

**Results:**

In total, 50 reports of observational studies were included; between 110,765 and 175,839 CE cases were considered for each sequela. The pooled proportion of CE cases that developed a sequela was 1.72% (95% CI 0.81–3.61; prediction interval [PI]: 0.03–47.65) for REA, 0.07% (0.03–0.16; PI: 0.003–1.59) for GBS, 0.22% (0.06–0.73; PI: 0.002–20.69) for Crohn's disease (CD), 0.35% (0.11–1.15; PI: 0.003–28.16) for ulcerative colitis (UC), and 4.48% (1.92–10.08; PI: 0.09–70.62) for IBS. The high between‐study heterogeneity could partially be explained by study size and design, the method of assessing sequelae, and the period between CE and sequelae onset. The quality of evidence was rated as moderate for GBS and UC, and low for REA, CD, and IBS.

**Conclusion:**

Updated estimates of the probability to develop sequelae after CE are provided, for CD and UC for the first time. However, uncertainty regarding the true probabilities remains, which is reflected in the broad PIs.

## Graphical abstract

Updated estimates on the probability to develop sequelae after a *Campylobacter* enteritis are made available in this meta‐analysis: The pooled proportions of *Campylobacter* enteritis cases that developed sequelae ranged between 0.07% for Guillain–Barré syndrome and 4.48% for irritable bowel syndrome.

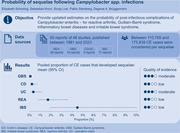

## INTRODUCTION

*Campylobacter* enteritis (CE) remains the most frequently reported zoonosis in humans in the European Union, with a notification rate of 41–66 confirmed cases per 100,000 population in recent years [1]. After an acute infection showing typical gastrointestinal symptoms, postinfectious complications can occur. Among the acknowledged sequelae of CE are reactive arthritis (REA) and Guillain–Barré syndrome (GBS). Furthermore, associations with inflammatory bowel diseases (IBD) and the irritable bowel syndrome (IBS) are discussed, although the exact mechanisms involved are not fully understood [2, 3].

To our knowledge, six (systematic) reviews and meta‐analyses dealing with the probability to develop sequelae after an infection with *Campylobacter* spp. have been performed so far: Two of them analyzed the occurrence of REA, GBS, IBD, and IBS [4, 5], whereas three studies focused on either REA [6, 7] or IBS [8]. Additionally, one scoping review was published [9], which included an overview of existing studies that describe different aspects of sequelae. The inclusion criteria differed somewhat among the reviews; the strictest criteria regarding the diagnosis of sequelae were applied by Svendsen et al. [8].

In previous meta‐analyses, results of the included single studies were pooled either by applying a random effects model using the inverse variance method [4, 8, 9] or by simply calculating a weighted mean [7]. Esan et al. [5] did not calculate any summary estimates due to the high heterogeneity among the included studies. The overall pooled proportion of CE cases that developed sequelae was 0.90%–2.86% for REA, 0.07% for GBS, and 0.12%–4.01% for IBS [4, 6–8]. For IBD, no summary estimate was calculated until now due to the small number of included studies.

Since then, new findings, especially regarding the development of IBD (i.e., Crohn's disease [CD] and ulcerative colitis [UC]) after CE, have been published. Moreover, instead of following the classic approach of pooling the reported proportions of CE cases that developed sequelae using the inverse variance method, generalized linear mixed models are now highly recommended for such proportional meta‐analyses [10, 11]. Besides, it is advised to compute and communicate the predicted range of the true proportions [12, 13, 14]. Therefore, the objective of this study was to update recent meta‐analyses regarding the probability to develop sequelae after CE by (i) including new studies and (ii) applying the recently recommended methodological approaches.

## METHODS

### Search strategy and eligibility criteria

In order to estimate the probabilities to develop REA, GBS, IBD, and/or IBS after CE, (i) previous meta‐analyses and systematic reviews were identified [4, 5, 6, 7, 8], (ii) all studies included in these publications were reviewed, and (iii) more recent studies were taken into account. This systematic review was performed in accordance with the PRISMA 2020 statement, the recently updated reporting guideline for the preferred reporting items for systematic reviews and meta‐analyses [15].

All studies included in the six previous meta‐analyses and reviews were retrieved and assessed for their eligibility. The scoping review by Pogreba‐Brown et al. [9] was the last comprehensive literature search conducted for the sequelae of interest in April 2018. More recent findings were identified through an additional search in MEDLINE via PubMed and Web of Science in July 2023. The search terms used were based on the previous searches and are listed in the Supporting Information Appendix. All results since January 2018 were screened.

In accordance with the previous meta‐analyses, study selection for this review was limited to observational studies (including outbreak investigations) that reported the number of confirmed cases of infections with *Campylobacter* spp. that developed REA, GBS, CD, UC, and/or IBS. Only studies with at least 15 CE cases were included; CE cases had to be confirmed by laboratory, clinical, and/or epidemiological findings. The type of diagnosis of sequelae was not restricted, that is, they could be either confirmed (i.e., diagnoses made by health professionals or according to medical records) or self‐reported.

### Screening and data extraction

Reports were independently screened by two reviewers (ES, SK). Disagreements among the reviewers were resolved by consensus. Data collection was performed by one author (ES) and was counterchecked by another author (SK). Data extraction included the study country, study design and duration, *Campylobacter* species, diagnosis type of CE, sample size, age and gender distribution, type and diagnosis of sequelae, follow‐up (i.e., period between CE and onset or diagnoses of sequelae), preexisting REA, GBS, IBD, and/or IBS, and the number of CE cases developing sequelae. In such cases, in which the number of sequelae cases was not reported, it was recalculated if possible.

### Risk of bias and quality of evidence assessment

Two reviewers (ES, SK) independently evaluated the risk of bias and the quality of evidence. The methodological quality of included reports was assessed using the JBI critical appraisal checklist for studies reporting prevalence data [16], in accordance with the systematic review by Esan et al. [5]. This tool consists of nine criteria addressing the appropriateness of sample frame, recruitment and study size, the adequate description and reporting of study subjects and settings, the appropriateness of statistical analysis, data coverage and response rate, as well as the validity and reliability of the outcome measurement. If no sample size calculation was reported, the adequate number of cases was recalculated as described in the Supporting Information Appendix. The appropriate handling of preexisting REA, GBS, IBD, and IBS cases (i.e., exclusion) is not part of the JBI critical appraisal checklist. Therefore, this potential source of bias was analyzed separately.

Additionally, the risk of bias due to missing, that is, not published, studies was evaluated. Established methods to assess publication or reporting bias, for example, Egger's test and funnel plots, might be inaccurate for proportional meta‐analyses. This is especially the case when extreme proportions are reported in the single studies [17]. As the probabilities that sequelae developed following CE were expected to be low or even zero, publication bias was assessed qualitatively instead [18]. However, there is no consensus regarding what a *positive*—and thus more likely published—result in studies reporting proportions should be. Iorio et al. [19] presumed that in prognostic studies, small studies reporting higher rates could suggest a selective publication. Following this assumption, the presence of such a pattern was evaluated in particular in the present study.

The quality of evidence was evaluated using the GRADE approach (Grading of Recommendations, Assessment, Development, and Evaluations) that distinguishes four quality levels: high, moderate, low, and very low confidence in the estimates [20]. As GRADE was initially developed to rate the quality of evidence of interventional studies in health care, it is not fully applicable to meta‐analyses of proportions. However, a guidance for the use of GRADE for prognostic studies was recently published [19]: Accordingly, starting from an initial rating of a high‐quality evidence, the following criteria were considered for down‐ or upgrading the rating: risk of bias, inconsistency of results, indirectness of evidence, imprecision, publication bias, large magnitude of effect and increase in events over time (dose–response gradient). The GRADE criterion relating to the effect of plausible residual confounding was not considered, as it does not apply to prognostic studies [19]. All decisions to down‐ or upgrade the quality of evidence were made by consensus.

### Statistical analysis

Meta‐analyses were performed by estimating random effects using generalized linear models (GLMMs) with a logit link [10, 21]. Additionally, a prediction interval (PI) was calculated based on Higgins et al. [13], which predicts the probable range of the proportion of sequela in CE cases in any (new) study.

From each of the included studies, one outcome was incorporated into the meta‐analyses; the main outcome was taken if studies reported multiple outcomes for different subgroups. For individual study results, Clopper–Pearson confidence intervals (CI) were calculated; the between‐study variance *τ* ^2^ was estimated using the maximum‐likelihood method. As a suitable test to assess heterogeneity in proportional meta‐analysis is lacking, the *I* ^2^ statistic was used [14]. *I* ^2^ is defined as the proportion of the between‐study variation due to true effects (and not due to sampling or estimation errors) and reflects the extent to which the CI of the individual studies do not overlap [12].

In line with the previous analyses, subgroups were analyzed, provided that at least three studies could be included for each group: (i) study size divided into studies with ≤1000 and >1000 CE cases, (ii) study design (prospective vs. retrospective design), (iii) infecting *Campylobacter* species, (iv) method of assessing the sequelae—either diagnoses confirmed by specialists and/or health professionals (including diagnoses according to medical records) or self‐reported diseases and/or symptoms, (v) follow‐up, that is, the period between CE and the onset or diagnoses of sequelae divided into less than 3 months, 3 months to less than 1 year, and 1 year and longer.

All analyses were performed in R 4.2.3 using the package meta [22].

## RESULTS

### Search results

All records of original studies included in the six previous meta‐analyses and reviews were retrieved (*n* = 71). Seventeen modeling studies that were described in the scoping review [9] were excluded, leaving 54 reports that were assessed for eligibility. Of these, 15 did not comply with the inclusion criteria and were also excluded.

The search strategy resulted in another 6846 records. After removing duplicates and records published before 2018, 765 records were screened for titles and abstracts. Of these, 23 reports were assessed for their eligibility, and 11 complied with the inclusion criteria [23, 24, 25, 26, 27, 28, 29, 30, 31, 32, 33].

**FIGURE 1:**
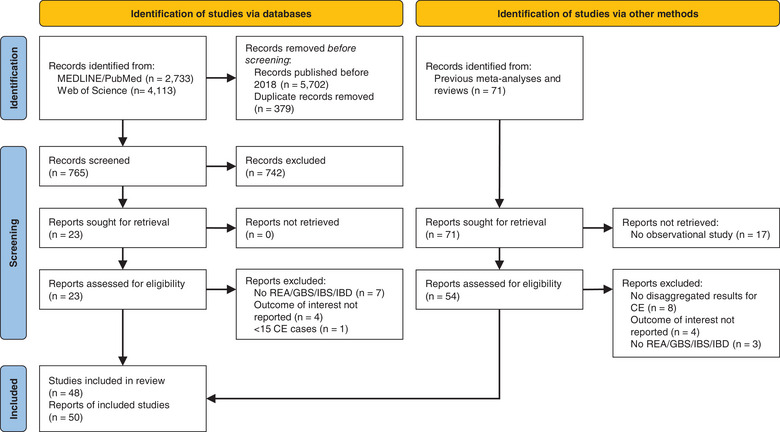
Preferred reporting items for systematic reviews and meta‐analyses (PRISMA) flow chart showing the study selection process. CD, Crohn's disease; CE, *Campylobacter* enteritis; GBS, Guillain–Barré syndrome; IBD, inflammatory bowel disease; IBS, irritable bowel syndrome; REA, reactive arthritis; UC, ulcerative colitis.

An overview of the study selection process is given in the PRISMA flow chart in Figure 1. The 27 reports that were assessed for their eligibility, but did not meet the inclusion criteria, are listed in the Supporting Information Appendix together with the reason for their exclusion.

A total of 50 reports of 48 studies were included in the meta‐analyses, reporting the probabilities to develop REA (*n* = 26), GBS (*n* = 14), CD (*n* = 5), UC (*n* = 5), and/or IBS (*n* = 17) following CE.

### Characteristics of the included studies

The majority of the included studies were cohort studies conducted in Europe, especially in the Scandinavian countries; the remaining studies were performed in Australia, Bangladesh, New Zealand, Taiwan, and the United States (Table 1). Ten outbreak investigations were also considered. Study directionality was slightly more often retrospective with 26 studies compared to 21 studies with a prospective design. The earliest report was published in 1981.

**TABLE 1:** Characteristics of studies included into meta‐analyses of the probability to develop sequela following *Campylobacter* enteritis (CE).

				CE				Sequelae			
References	Country	Study design	Study duration	Species	Diagnosis of CE	Age (years)	Female (%)	Sequelae	Diagnosis of sequelae	Follow‐up^a^	Preexisting sequelae
Baker et al. [34]	New Zealand	Retrospective cohort (hospitalized CE cases), hospital records	1995–2010	*Campylobacter* spp.	Laboratory or epidemiologically confirmed (medical records)	0 to ≥80, median 41	NR	GBS	Confirmed, hospitalized cases (medical record)	0–28 days	None
Berumen et al. [28]	USA	Retrospective cohort	2011–2019	*C. jejuni, C. coli*, others, undetermined	Laboratory confirmed	18–80	44	IBS	Self‐reported disease status (IBS according to Rome III criteria)	6–9 months	Cases excluded
Breen‐Lyles et al. [30]	USA	Retrospective cohort	2019–2022	*C. jejuni*	Laboratory confirmed	NR	50	IBS	Self‐reported disease status (IBS according to Rome IV criteria)	6–9 months	Cases excluded
Bremell et al. [35]	Sweden	Prospective outbreak (foodborne) investigation	1981–1986	*C. jejuni*	Laboratory confirmed	NR, adults	56	REA	Self‐reported disease status (according to author's definition of REA)	10 days to 8 months	None
Doorduyn et al. [36]	The Netherlands	Prospective case‐control study	2002–2005	*Campylobacter* spp.	Laboratory confirmed	NR	NR	REA, GBS	Self‐reported diagnoses (author's definition of REA and GBS not specified)	Up to 3.8 years	No information
Dunlop et al. [37]	United Kingdom	Prospective cohort	1999–2002	*C. coli, C. jejuni*	Laboratory confirmed	18–75	NR	IBS	Self‐reported disease status (IBS according to Rome I criteria)	3 months	Cases excluded
Eastmond et al. [38]	United Kingdom	Retrospective outbreak (foodborne) investigation	Outbreak in 1979	*C. jejuni*	Laboratory confirmed	NR	NR	REA	Confirmed (medical record)	3 months	None
Esan et al. [23]	United Kingdom	Retrospective cohort, health records	2000–2015	*Campylobacter* spp.	Laboratory confirmed (medical record)	0 to ≥65	46	REA, GBS, CD, UC, IBS	Confirmed (medical record)	12 months	Cases excluded
Gardner et al. [39]	USA	Prospective outbreak (foodborne) investigation	2008	*C. jejuni*	Laboratory or clinico‐epidemiologically confirmed	1–79, median 47	NR	GBS	Confirmed (by specialist)	9 days	No information
Gilpin et al. [24]	New Zealand	Prospective outbreak (waterborne) investigation	2016	*C. jejuni*	Laboratory or clinico‐epidemiologically confirmed	<5 to ≥60	52	GBS	No information	NR	No information
Gumpel et al. [40]	United Kingdom	Retrospective cohort, medical records	1978	*Campylobacter* spp.	Laboratory confirmed	≥5	NR	REA	Confirmed (medical record)	NR	No information
Hannu et al. [41]	Finland	Prospective case‐control study	1997–1998	*C. jejuni, C. coli*, undetermined	Laboratory confirmed	1–91, mean 37.1	59	REA	Confirmed (by specialist)	2 months	Not considered sequelae
Hannu et al. [42]	Finland	Prospective outbreak (waterborne) investigation	2000	*C. jejuni*	Clinico‐epidemiologically confirmed	NR	NR	REA	Confirmed (by specialist)	3 months	Not considered sequelae
Helms et al. [43]	Denmark	Retrospective cohort, national registries	1991–1999	*Campylobacter* spp.	Laboratory confirmed	0–102, median 26	52	REA, GBS, IBD, IBS	Confirmed, hospitalized cases (medical record)	Up to 1 year	Not considered sequelae
Jalanka et al. [33]	United Kingdom	Prospective cohort	2014–2016	*C. jejuni*.	Laboratory confirmed	>18	52	IBS	Self‐reported disease status (IBS according to Rome III criteria)	3 months	Cases excluded
Jess et al. [44]	Denmark	Retrospective cohort, national registries	1992–2008	*Campylobacter* spp.	Laboratory confirmed	0 → 60	48	CD, UC	Confirmed (medical record)	Up to 16 years	Cases excluded
Johnsen et al. [45]	Norway	Retrospective cohort	1980–1981	*C. jejuni*	Laboratory confirmed	NR	35	REA	Confirmed (by specialist/medical record)	1–17 days	No information
Kosunen et al. [46]	Finland	NR, medical records	1978–1979	*C. jejuni*	Laboratory confirmed	NR	NR	REA	Confirmed (by specialist/medical record)	4–28 days	No information
Locht and Krogfelt [47]	Denmark	Retrospective cohort	1997–2000	*C. coli, C. jejuni*	Laboratory confirmed	18–76	57	REA	Self‐reported disease status (according to author's definition of probable REA)	4 weeks	Not considered sequelae
McAllister et al. [31]	Australia	Retrospective outbreak (foodborne) investigation	2022	*C. jejuni*	Laboratory or epidemiologically confirmed	18–69, median 29	45	GBS, IBS	Self‐reported disease status (author's definition of probable GBS/IBS not specified)	NR	No information
McCarthy et al. [48]	Sweden	Retrospective outbreak (waterborne) investigation	1980, 1994, 1995	*C. jejuni*	Laboratory or epidemiologically confirmed	NR	NR	GBS	Confirmed, hospitalized cases (medical record)	6 months	Not considered sequelae
McCarthy and Giesecke [49]	Sweden	Retrospective cohort, national registries	1987–1995	*C. jejuni*	Laboratory confirmed	0 to ≥85	NR	GBS	Confirmed, hospitalized cases (medical record)	6 months	Cases excluded
Melby et al. [50]	Norway	Retrospective outbreak (waterborne) investigation	NR	*C. jejuni*	Laboratory or epidemiologically confirmed	1 to ≥85	48	REA	Self‐reported disease status (author's definition of REA not specified)	6 weeks	No information
Melby et al. [51]	Norway	Retrospective outbreak (waterborne) investigation	1988	*C. coli, C. jejuni*	Laboratory or epidemiologically confirmed	All ages	NR	REA	Self‐reported disease status (author's definition of REA not specified)	NR	No information
Nielsen et al. [52]	Denmark	Prospective cohort	2009–2010	*C. coli, C. concisus, C. jejuni*	Laboratory confirmed	>18	54	IBS	Self‐reported disease status (according to author's definition of IBS), confirmed in hospitalized cases (medical record)	6 months	Cases excluded
Nielsen et al. [27]	Denmark	Retrospective cohort, national registries	2009–2018	*C. coli, C. concisus, C. jejuni*	Laboratory confirmed	≥15	52	CD, UC	Confirmed (medical record)	Median of 5.6 years	Cases excluded
Petersen et al. [53]	Denmark	Retrospective cohort (hospitalized CE cases), hospital records	1991–1993	*C. coli, C. jejuni*	Laboratory confirmed	All ages, median 21	NR	REA	Confirmed, hospitalized cases (medical record)	NR	None
Pitkänen et al. [54]	Finland	Prospective cohort, hospital records	1978–1980	*C. jejuni*	Laboratory confirmed	11–76	46	REA	Confirmed (medical record) and self‐reported disease status (author's definition of REA not specified)	NR	Case excluded
Pitkänen et al. [55]	Finland	Prospective cohort (hospitalized CE cases), hospital records	1978–1981	*C. jejuni*	Laboratory confirmed	0–89	47	REA	Confirmed (medical record) and self‐reported disease status (author's definition of REA not specified)	1–3 weeks	No information
Pönkä et al. [56]	Finland	Prospective cohort (outpatients)	1978–1981	*C. jejuni*	Laboratory confirmed	All ages	NR	REA	Self‐reported disease status (author's definition of arthritis not specified)	NR	No information
Porter et al. [57, 58]	USA	Retrospective cohort (US military personnel), medical records	1998–2009	*Campylobacter* spp.	Clinically confirmed (medical record)	Adults, median 29	15	REA, IBS^b^	Confirmed (medical record)	6 months (REA)/median of 3.8 years (IBS)	No information (REA)/cases excluded (IBS)
Rahman et al. [59]	Bangladesh	Prospective cohort (hospitalized CE cases)	2014–2016	*Campylobacter* spp.	Clinically and laboratory confirmed	≥18	NR	IBS	Self‐reported disease status (IBS according to Rome III criteria)	12 months	Cases excluded
Rees et al. [60]	USA	Prospective cohort	1998–2000	*Campylobacter* spp.	Laboratory confirmed	NR	NR	IBS	Self‐reported diagnoses	3 months	None
Scallan Walter et al. [26]	USA	Retrospective cohort, health insurance data	2010–2014	*Campylobacter* spp.	Clinically confirmed (medical record)	<1 to 64	49	IBS	Confirmed (medical record)	Up to 5 years	Cases excluded
Scallan Walter et al. [25]	USA	Retrospective cohort, health insurance data	2004–2013	*Campylobacter* spp.	Clinically confirmed (medical record)	0–102, median 43	51	GBS	Confirmed (medical record)	8 weeks	Not considered as sequelae
Schiellerup et al. [61]	Denmark	Prospective cohort	2002–2003	*Campylobacter* spp.	Laboratory confirmed	≥18	NR	REA	Self‐reported disease status (according to author's definition of reactive joint pain)	4 weeks	Cases excluded
Schönberg‐Norio et al. [62]	Finland	Cross sectional/prospective cohort^c^	2002	*C. jejuni*	Laboratory confirmed	1–88, median 50	48	REA	Confirmed (medical record)	2 months	Not considered sequelae
Schorling et al. [32]	Germany	Retrospective cohort, health insurance data	2017–2019	*Campylobacter* spp.	Clinically confirmed (medical record)	0–97, median 47	51	REA, GBS, CD, UC, IBS	Confirmed (medical record)	Up to 36 months	Not considered sequelae
Short et al. [63]	United Kingdom	Prospective cohort, hospital records	1979–1980	*C. jejuni*	Laboratory confirmed	NR	NR	REA	Confirmed (medical record)	6 weeks	No information
Spence and Moss‐Morris [64, 65]	New Zealand	Prospective cohort	2002–2003	*Campylobacter* spp.	Laboratory confirmed	>16	NR	IBS	Self‐reported disease status (IBS according to Rome I/II criteria)	6 months	Cases excluded
Spiller et al. [66]	United Kingdom	Prospective cohort	NR	*Campylobacter* spp.	Laboratory confirmed	≥20	NR	IBS	Self‐reported disease status (IBS according to Rome I criteria)	1 year	No information
Tam et al. [67]	United Kingdom	Retrospective cohort, health records	1991–2001	*Campylobacter* spp.	Clinically confirmed (medical record)	NR	NR	GBS	Confirmed (medical record)	60 days	Not considered sequelae
Ternhag et al. [68]	Sweden	Retrospective cohort	1997–2004	*Campylobacter* spp.	Laboratory confirmed	0–99, mean 37	47	REA, GBS, CD, UC, IBS	Confirmed, hospitalized cases (medical record)	3 months (GBS)/1 year	No information (except for UC: cases excluded)
Thornley et al. [69]	United Kingdom	Prospective cohort	1997	*Campylobacter* spp.	Laboratory confirmed	≥18	NR	IBS	Self‐reported disease status (IBS according to Rome I criteria)	6 months	Not considered sequelae
Townes et al. [70]	USA	Prospective cohort	2002–2004	*Campylobacter* spp.	Laboratory confirmed	>1, median 35	47	REA	Confirmed (by specialist) after self‐reported disease status	Median of 43 days	Not considered sequelae
Walker et al. [29]	New Zealand	Retrospective outbreak (waterborne) investigation	2016	*Campylobacter* spp.	Laboratory confirmed	1–96, median 47	45	REA	Self‐reported disease status (according to author's definition of probable REA)	Up to 12 weeks	Not considered sequelae
Wang et al. [71]	Taiwan	Retrospective cohort, hospital records	2000–2006	*C. coli, C. jejuni*	Laboratory confirmed	0 to <18, mean 5.5	29	GBS	Confirmed (medical record)	NR	No information
Zia et al. [72]	United Kingdom	Retrospective cohort	1999	*C. jejuni*	Laboratory confirmed	≥16, mean 48	56	REA	Self‐reported disease status (author's definition of reactive joint swelling not specified)	Up to 4 months	Not considered sequelae

Abbreviations: CD, Crohn's disease; GBS, Guillain–Barré syndrome; IBD, inflammatory bowel disease; IBS, irritable bowel syndrome; NR, not reported; REA, reactive arthritis; UC, ulcerative colitis.

^a^ The (observation) period between CE and the onset or diagnoses of sequelae.

^b^ The number of IBS cases among CE cases was calculated according to the relative risk given in the study [68].

^c^ Cross‐sectional study according to the authors; equivalent to a prospective cohort as in the other included studies.

The study size ranged between 15 and 57,425 CE cases; in 15 studies, >1000 CE cases were reported. Enrolment of CE cases differed between the studies in terms of age (15 studies focused on adults and one study on children) and presumed severity of CE (in four studies limited to hospitalized, i.e., severe, cases).

In 34 studies, all CE cases were laboratory confirmed, whereas the remaining studies used a clinical, laboratory, and/or epidemiological confirmation of CE. The *Campylobacter* species was specified in 25 studies, whereas 23 studies generally reported *Campylobacter* spp. or a mix of several *Campylobacter* species.

The minimum follow‐up period was 9 days with a maximum of up to 16 years; in eight studies, no information was available. Diagnoses of sequelae were confirmed by specialists and/or health professionals in 27 of the 48 included studies and were restricted to hospitalized cases in 6 studies.

### Pooled proportions of sequelae following *Campylobacter* infections

**FIGURE 2:**
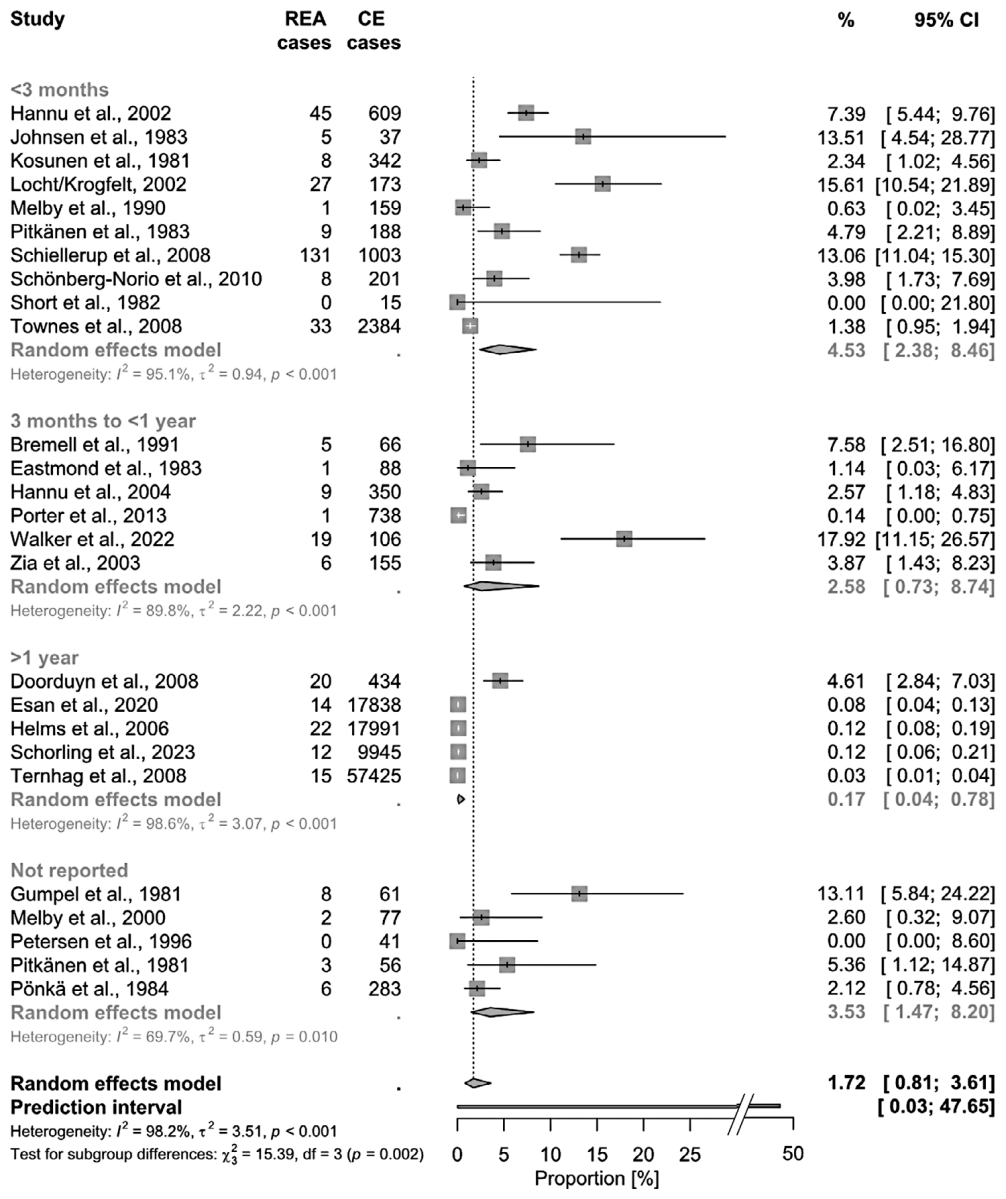
Forest plot of studies reporting the proportion of *Campylobacter* enteritis (CE) cases that developed reactive arthritis (REA), stratified by follow‐up period. The results of all subgroup analyses are displayed in the Supporting Information Appendix.

**FIGURE 3:**
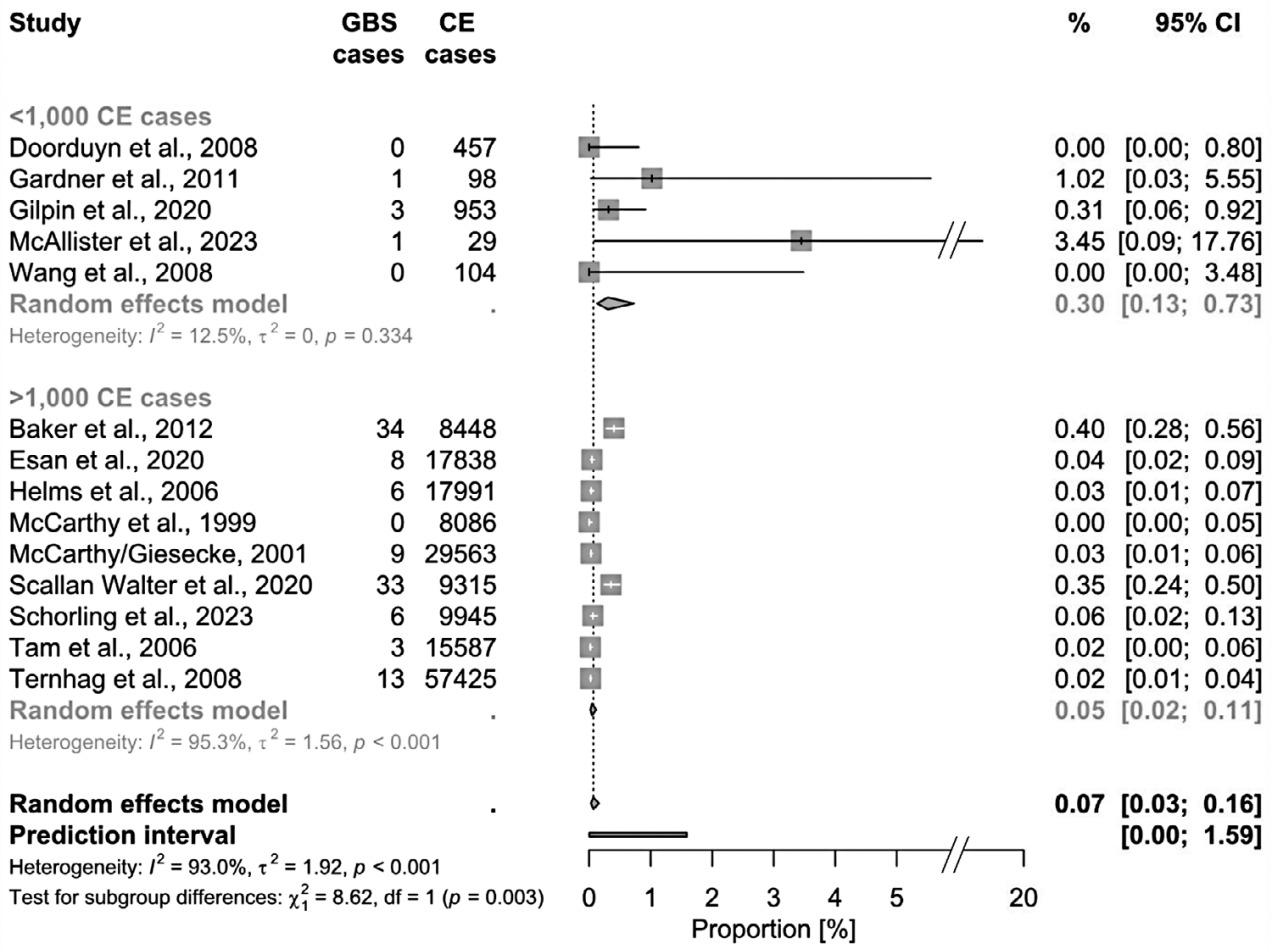
Forest plot of studies reporting the proportion of *Campylobacter* enteritis (CE) cases that developed Guillain–Barré syndrome (GBS), stratified by study size. The results of all subgroup analyses are displayed in the Supporting Information Appendix.

**FIGURE 4:**
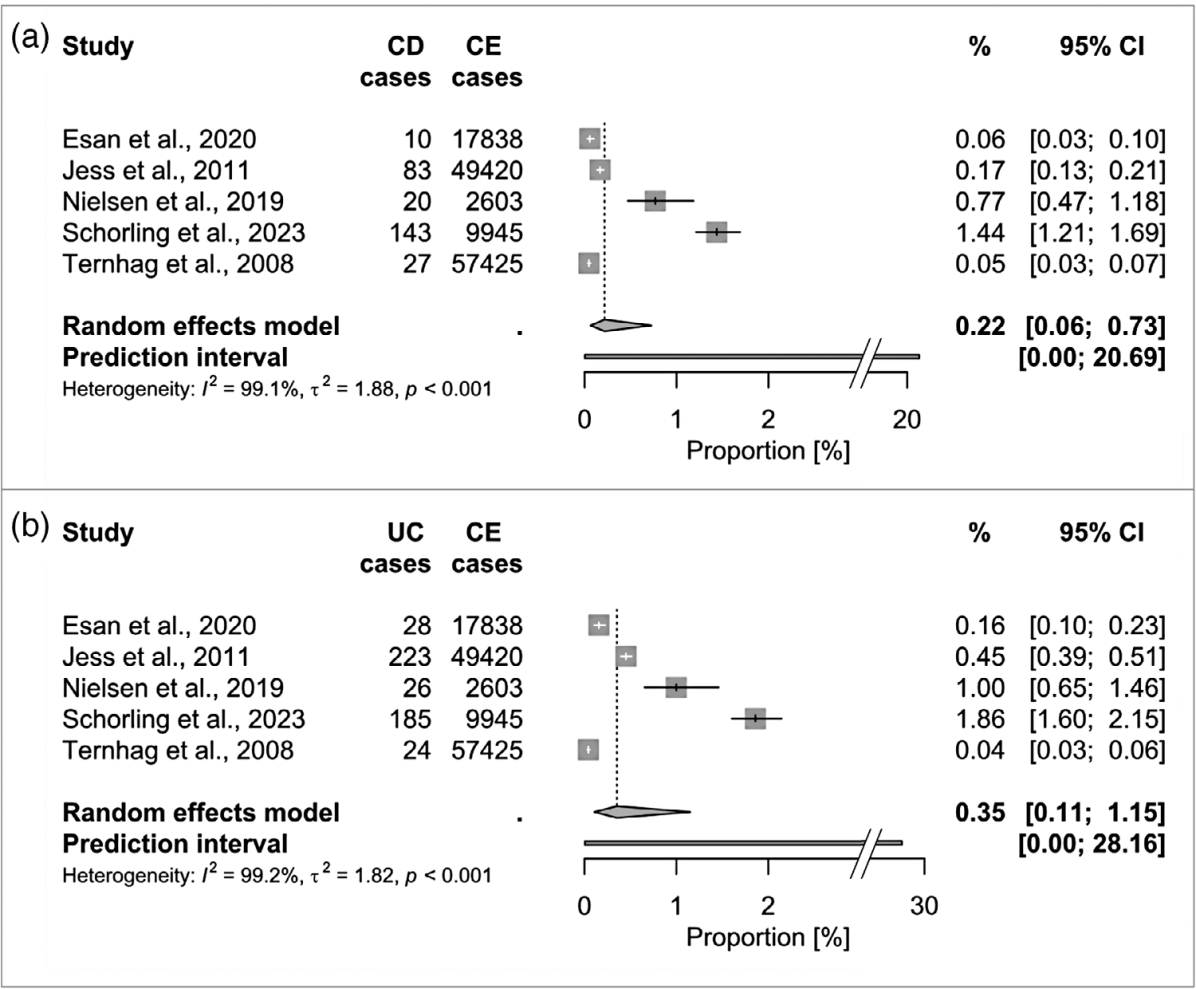
Forest plot of studies reporting the proportion of *Campylobacter* enteritis (CE) cases that developed inflammatory bowel diseases: (A) Crohn's disease (CD), (B) ulcerative colitis (UC).

**FIGURE 5:**
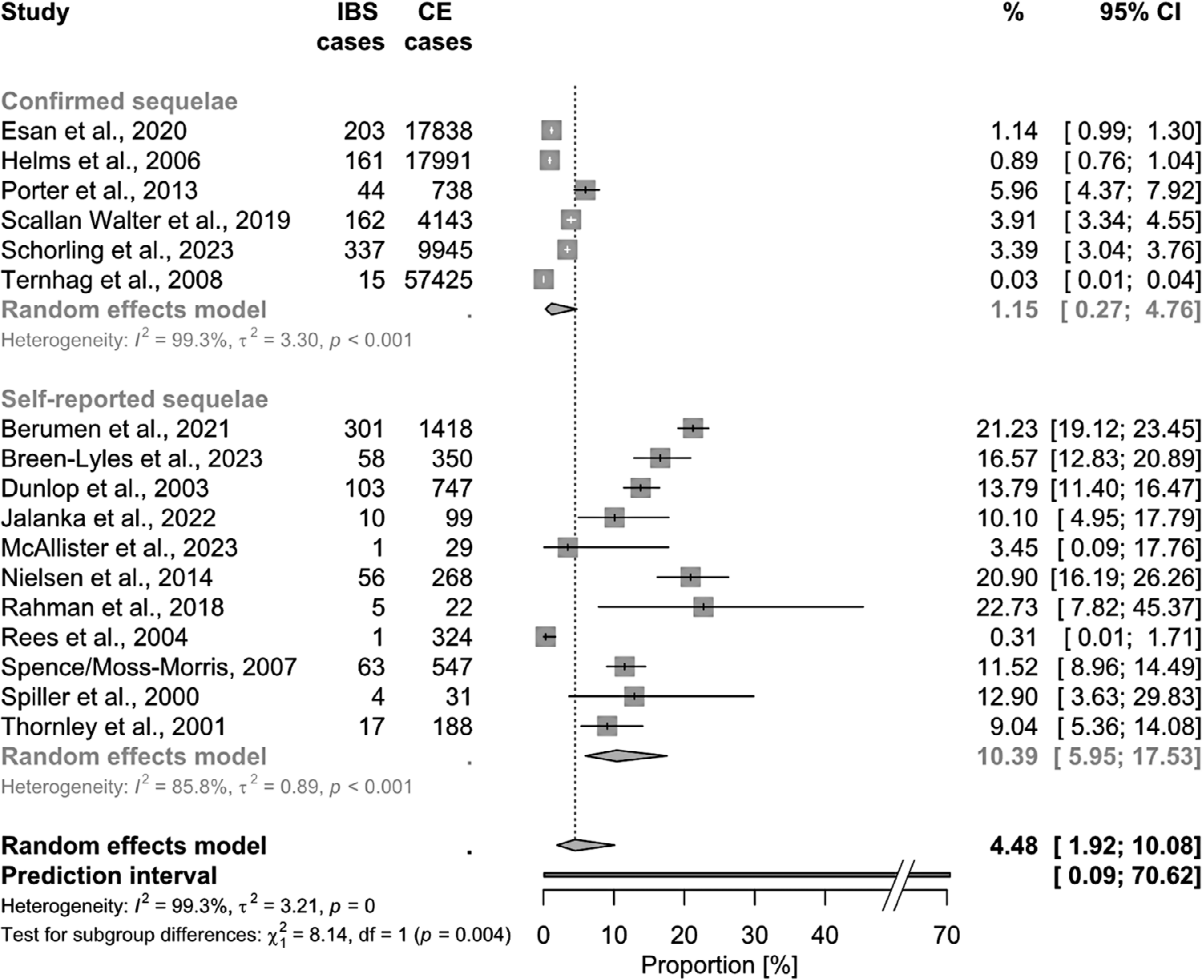
Forest plot of studies reporting the proportion of *Campylobacter* enteritis (CE) cases that developed irritable bowel syndrome (IBS), stratified by sequelae diagnosis. The results of all subgroup analyses are displayed in the Supporting Information Appendix.

In total, for each sequela, at least 110,765 CE cases (screened for REA) and up to 175,839 CE cases (for GBS) were considered. The pooled proportion of CE cases that developed REA was 1.72% (95% CI 0.81–3.61, Figure 2). The associated PI was 0.03%–47.65%. For GBS, the proportion was 0.07% (95% CI 0.03–0.16; PI: 0.003%–1.59%, Figure 3). Regarding IBD, the pooled proportions were slightly higher for UC with 0.35% (95% CI 0.11–1.15; PI: 0.003%–28.16%) compared to CD with 0.22% (95% CI 0.06–0.73; PI: 0.002%–20.69%, Figure 4). The highest pooled estimate was found for IBS with 4.48% (95% CI 1.92–10.08; PI: 0.09%–70.62%, Figure 5).

The between‐study heterogeneity according to the *I* ^2^ statistic was high (>90% in all meta‐analyses) but was lower in some subgroups depending on study size and design, sequelae diagnosis, as well as follow‐up period (results of subgroup meta‐analyses are presented in the Supporting Information Appendix). However, a considerable reduction of the heterogeneity was only seen for GBS, with an *I* ^2^ statistic of 0.0 to 12.5% in studies (i) with a follow‐up of 3 months to ≥1 year, (ii) with a prospective design, or (iii) with ≤1000 CE cases.

Subgroup analyses revealed differing probabilities of sequelae depending on study size: Higher proportions of REA, GBS, and IBS were seen in studies with ≤1000 compared to >1000 CE cases (Figure 3). The most pronounced difference was observed for REA: Studies with smaller sample sizes reported a 12 times higher probability to develop REA than larger studies (3.61% vs. 0.29%).

REA and IBS seemed to occur more often in cases, in which diagnoses or symptoms were self‐reported, than in cases, in which diagnoses were confirmed by specialists and/or health professionals (pooled estimate of 5.5% vs. 0.9% for REA and 10.4% vs. 1.1% for IBS, respectively; Figure 5). For GBS, no subgroup analysis was performed as only two studies analyzed self‐reported GBS, and in one report, the information about the diagnosis type was missing.

Moreover, the proportion of CE cases that developed REA or GBS decreased with increasing follow‐up time range: For REA, the proportion was drastically reduced to 0.17% if the period between the onset of CE and the diagnosis of sequelae was ≥1 year (Figure 2); for GBS, the sharp decline already occurred after 3 months.

Regarding the study design, the proportion of CE cases that developed sequelae tended to be higher in prospective studies than in retrospective studies; however, this trend was only statistically significant for GBS.

In order to perform subgroup analyses by *Campylobacter* species, additional species‐specific outcomes were included (forest plots stratified by *Campylobacter* species are provided in the Supporting Information Appendix). The number of reports presenting results for species other than *Campylobacter jejuni* was limited. Therefore, only comparisons between *C. jejuni* and the mix of different *Campylobacter* species (including combinations of *C. jejuni/Campylobacter coli, Campylobacter* spp. in general, and undetermined *Campylobacter* species) were analyzed but did not show any statistically significant differences (Supporting Information Appendix).

For CD and UC, no subgroup analyses were performed due to the small number of included studies.

### Risk of bias and quality of evidence

Of the included 49 reports assessed for risk of bias, 47 fulfilled more than 50% of the criteria according to the JBI critical appraisal checklist; two reports met the complete set of criteria (the evaluation of each study is provided in the Supporting Information Appendix; the report by Moss‐Morris and Spence [64] was not evaluated separately). Overall, 76.1% of the criteria were appropriately addressed in the included reports; the proportion was lower in reports of REA with 75.3% and was highest for reports of CD and UC, which fulfilled 94.0% of the criteria.

The most critical aspect was the adequacy of the sample size. Overall, only 11 reports fulfilled this criterion; compliance was especially low in reports regarding REA and GBS: For REA, the corresponding reports particularly often included a small sample size; thirteen of the 26 reports included <200 CE cases. In contrast, studies analyzing postinfectious GBS required approximately 20,000 CE cases due to the low expected prevalence of GBS after CE (as explained in the Supporting Information Appendix), which was only achieved in two out of 14 reports.

Additionally, the handling of preexisting sequelae was analyzed. Only all reports on UC provided details regarding the occurrence and exclusion of preexisting illnesses, whereas the information was missing in 18% (IBS) to up to 43% (GBS) of the included reports on the other sequelae. In order to analyze the potential impact of the missing information on the pooled estimates, a separate subgroup analysis was performed for REA, GBS, and IBS, but it did not reveal statistically significant differences (no or excluded preexisting cases vs. no information, *p* > 0.2).

According to the results of the subgroup analyses, studies with smaller sample sizes reported higher proportions of sequelae, which could indicate a potential publication bias [19]. However, in the case of REA and GBS, both especially low and especially high proportions of sequelae, respectively, were observed in the smallest studies (i.e., ≤110 CE cases). For IBS, the comparatively low proportions in larger studies were probably highly influenced by the diagnosis type, as in the majority of the studies with >1000 CE cases, sequelae were confirmed by specialists and/or health professionals and not self‐reported (Figure 5). Although a publication bias cannot be completely ruled out, it seems to be unlikely.

The quality of evidence was rated as moderate for GBS and UC and as low for REA, CD, and IBS: Confidence in the estimates of REA, GBS, CD, and IBS was downgraded by one level due to study limitations, as some of the included reports did not clarify if preexisting cases were identified and excluded (as mentioned above). Inconsistency of results led to a downgrade by one level for all sequelae except for GBS. Indirectness, imprecision, publication bias, magnitude of effect, or a dose–response gradient did not alter the rating of the quality of evidence.

## DISCUSSION

With this systematic review and meta‐analysis, updated estimates on the probability to develop REA, GBS, and IBS following *Campylobacter* infections are presented. Additionally, for the first time, we were able to estimate pooled probabilities of CD and UC by including three new studies published between 2019 and 2023 reporting the probability of IBD after CE.

In contrast to previous meta‐analyses on the probability of sequelae [4, 6, 7, 8], we estimated random effects by using a GLMM instead of the inverse variance method. GLMM seems to be more suitable to pool proportions when the within study variance has large uncertainties, for example, due to small sample sizes or rare or zero events [11, 21], which was the case in some of the studies. Together with the inclusion of new studies, this led to pooled estimates that did not strongly differ from the results of previous analyses with similar inclusion criteria [4, 6]. However, if we used the classic approach of meta‐analysis, the mean probabilities of the update were somewhat higher than those previously reported (2.06% for REA, 0.11% for GBS, and 4.79% for IBS).

Previous meta‐analyses were amended by retrieving and reanalyzing all studies included in them. However, 15 reports were excluded, as they did not report disaggregated results for CE or did not report the number of CE cases that developed the sequelae of interest. Although two of them were considered eligible by others [4, 5], we decided to exclude the studies by Saps et al. [73] and Uotila et al. [74], as they did not report the development of IBS and REA after CE, but that of *functional gastrointestinal disorders* and *arthritis like symptoms* instead. Similarly, the proportions of *chronic intestinal diseases* reported by Doorduyn et al. [36] and of *joint pain* reported by Rees et al. [60] were not taken into account in the meta‐analyses for IBS and REA, although they were included in two previous analyses [4, 7].

The search for more recent studies was performed by only analyzing two databases (MEDLINE via PubMed and Web of Science). Therefore, we cannot rule out the possibility that other potentially eligible studies might have been missed. However, all studies included in the previous meta‐analyses—except one [59]—could be found. Rahman et al. [59] analyzed the occurrence of IBS following infectious diarrhea in general, and the *Campylobacter*‐specific proportions were mentioned only briefly. This study was included in the meta‐analysis by Svendsen et al. [8], which also considered a broader spectrum of pathogens.

As observed in earlier meta‐analyses [4, 6, 7], the between‐study heterogeneity according to the *I* ^2^ statistic was high for all sequelae. Study size and design, the method of assessing sequelae, and the follow‐up period could explain to some extent the heterogeneity, which is in line with previous analyses [4, 5, 6]. The *I* ^2^ statistic was also influenced by sampling errors, which were minimized in studies with a high number of observed CE cases [12, 19], whereas the broad and overlapping CI in reports with ≤1000 CE cases led to a lower *I* ^2^ (as shown for GBS in Figure 3). Similarly, the majority of studies reporting either REA cases after a follow‐up of ≥1 year (Figure 2) or confirmed IBS cases (Figure 5) had a large sample size and narrow CI. Therefore, the *I* ^2^ was very high in these subgroups, which means that there was almost no overlap between the CI. Nevertheless, the reported proportions of the single studies were rather consistent.

By including reports on the proportion of sequelae following CE with no or few restrictions regarding the year and country of dissemination, the study design and the diagnosis type of sequelae, the available evidence was summarized. These rather broad eligibility criteria also increased the between‐study heterogeneity and presumed inconsistency of results. However, as stated by Barker et al. [14], some heterogeneity between incidence estimates is to be expected due to differences in time and place of study conduct, which does not necessarily imply inconsistencies. However, as a consequence, the PIs were rather broad for all sequelae, but especially for IBS and REA.

Heterogeneity was also observed regarding the methodological quality of the included reports. Although we used the same tool for the critical appraisal of included reports as Esan et al. [5], our evaluation slightly differed in some points (as indicated in the Supporting Information Appendix): We judged the coverage of the identified sample (criterion 5) more often as *unclear*, as response rates among subgroups were seldom reported, and the presented characteristics of participants did not always allow for an assessment. Further discrepancies in the critical appraisal can be explained as follows: (i) We used the most recent pooled proportions of sequelae [4, 6] in the recalculation of the adequate sample size (criterion 3), (ii) we evaluated the description of study subjects explicitly with regard to the CE cases (criterion 4), and (iii) we defined diagnoses of sequelae as reliable if they were made by (trained) specialists (criterion 7b).

As mentioned above, this update of meta‐analyses has some limitations. The pooled estimates should therefore be interpreted in this context. The high heterogeneity between studies could not be fully explained by subgroup analyses and could limit the generalizability of the estimates. Study limitations and inconsistency of results led to a downgrade of the quality of evidence to a moderate or low level. Therefore, to incorporate the high heterogeneity and uncertainty into the analyses, the PIs seem to be the adequate—or at least a more conservative [14]—way to evaluate the probabilities to develop sequelae following CE.

Uncertainty also remains regarding the causality between IBD/IBS and CE. Although epidemiologic evidence suggests an association, the role of *Campylobacter* spp. in the pathogenesis of CD, UC, and IBS is not fully understood. It is hypothesized that a dysregulated immune response, changes in the gut microbiota, increased intestinal permeability, and (epi)genetic host factors contribute to the development of both postinfectious IBD and IBS [3, 75, 76, 77].

Despite these limitations, the systematic review and meta‐analyses provide new estimates—in the case of IBD for the first time—of the probability to develop sequelae after CE, following the latest recommendations on meta‐analyses of proportions. The utility of updated pooled proportions is evident as more and more studies reporting the health and/or economic burden of campylobacteriosis, including sequelae, have been published in recent years, as listed by Pogreba‐Brown et al. [9]. In many of these modeling studies, the uncertainty about the true probabilities of sequelae following CE is included via assumptions of their distribution functions, as is done, for example, in the BCoDE toolkit by the European Centre for Disease Prevention and Control based on Dutch data [78]. In a recent estimate of the health burden of campylobacteriosis in Germany, the lowest and highest proportions according to published studies were used as the minimum and maximum values in a PERT distribution [79]. Instead of using the range of reported probabilities, we propose the integration of the PIs in probabilistic modeling approaches, as they will better reflect the remaining uncertainty associated with the probability of sequelae following *Campylobacter* infections.

## CONCLUSION

The health burden of CE remains high and is considerably increased by the occurrence of postinfectious complications. In the present study, the existing evidence on sequelae following confirmed CE was summarized. According to the updated probabilities provided, IBS is still the most common sequelae following CE—although the causality is still under discussion—followed by REA. IBD and GBS are rather rare complications of CE.

The results of this study can contribute to improve the assessment of the health (and economic) burden of campylobacteriosis—both on a national and international level—through the provision of evidence‐based data. Such estimates are an important source of data for policy making in public health. However, the quality of evidence was rated as moderate or low due to risk of bias and inconsistencies. Therefore, the PIs seem to be particularly useful, as they incorporate the remaining uncertainty associated with the pooled estimates.

## AUTHOR CONTRIBUTIONS

*Formal analysis; methodology; writing—original draft; writing—review and editing*: Elisabeth Schorling. *Formal analysis; methodology; writing—review and editing*: Sebastian Knorr. *Conceptualization; project administration; writing—review and editing*: Sonja Lick. *Writing—review and editing; Supervision*: Pablo Steinberg. *Conceptualization; project administration; supervision; writing—review and editing*: Dagmar Adeline Brüggemann.

## CONFLICT OF INTEREST STATEMENT

The authors have declared that no conflicts of interest exist.

## FUNDING INFORMATION

The authors received no specific funding for this work.

## ETHICS STATEMENT

N/A: Secondary analysis; no human participants involved.

## Supporting information

Supporting Information

## Data Availability

No new data were created or analyzed in this study that could be shared; the data that support the findings of this review and meta‐analysis were extracted from the included studies listed in Table 1.
