# Supplementary material for: Probability of sequelae following Campylobacter spp. infections: Update of systematic reviews and meta‐analyses
Source: Public Health Chall. 2023 Dec 20;2(4):e145. doi: 10.1002/puh2.145 (PMC12039688; doi:10.1002/puh2.145)
Supplement: Supplementary file 1 — Supporting Information [file PUH2-2-e145-s001.pdf]

Supplementary materials

Probability of sequelae following *Campylobacter spp.* infections: Update of systematic reviews and meta-analyses

Elisabeth Schorling, Sebastian Knorr, Sonja Lick, Pablo Steinberg and Dagmar A. Brüggemann

Table 1: Search strategy

	**Query**
MEDLINE via PubMed	("Campylobacter"[MeSH Terms] OR "Campylobacter Infections"[MeSH Terms] OR "campylobact*"[All Fields]) AND ("sequel*"[Title/Abstract] OR "long-term"[Title/Abstract] OR "chronic"[Title/Abstract] OR "complication*"[Title/Abstract] OR "guillain*"[Title/Abstract] OR "Guillain-Barre Syndrome"[MeSH Terms] OR "arthritis"[Title/Abstract] OR "arthritis, reactive"[MeSH Terms] OR "Inflammatory Bowel Diseases"[MeSH Terms] OR "IBD"[Title/Abstract] OR "crohn*"[Title/Abstract] OR "ulcerative colitis"[Title/Abstract] OR "Irritable Bowel Syndrome"[MeSH Terms] OR "IBS"[Title/Abstract] OR "Irritable Bowel Syndrome"[Title/Abstract])
Web of Science	(TS=(Campylobacter)) AND TS=("sequelae" OR "long-term" OR "chronic" OR "complication*" OR ("guillain" AND "barre") OR "arthritis" OR "reactive arthritis" OR "Inflammatory Bowel" OR "IBD" OR "crohn" OR "ulcerative colitis" OR "irritable bowel syndrome" OR "IBS")

Search terms based on previous searches conducted [1–5].

Table 2: Reports assessed for eligibility that were not included in the review

Report	Record identified from	Reason for exclusion
Barrett et al., 2018 [6]	databases	outcome of interest not reported
Borgaonkar et al., 2006 [7]	previous meta-analyses and reviews	no results for *Campylobacter* enteritis
Emberland et al., 2022 [8]	databases	no REA/GBS/IBS/IBD
Friesema et al., 2022 [9]	databases	no REA/GBS/IBS/IBD
Garg et al., 2006 [10]	previous meta-analyses and reviews	no REA/GBS/IBS/IBD
Gradel et al., 2009 [11]	previous meta-analyses and reviews	no results for *Campylobacter* enteritis
Hossain et al., 2023 [12]	databases	no REA/GBS/IBS/IBD
Iacob et al., 2020 [13]	databases	<15 CE cases
Laine et al., 2014 [14]	previous meta-analyses and reviews	no results for *Campylobacter* enteritis
Lee et al., 2022 [15]	databases	outcome of interest not reported
Li et al., 2020 [16]	databases	no REA/GBS/IBS/IBD
Marshall et al., 2010 [17]	previous meta-analyses and reviews	no results for *Campylobacter* enteritis
Neal et al., 1997 [18]	previous meta-analyses and reviews	no results for *Campylobacter* enteritis
Neal et al., 2002 [19]	previous meta-analyses and reviews	no results for *Campylobacter* enteritis
O'Connor et al., 2020 [20]	databases	no REA/GBS/IBS/IBD
Omarova et al., 2023 [21]	databases	outcome of interest not reported
Parry et al., 2003 [22]	previous meta-analyses and reviews	no results for *Campylobacter* enteritis
Pierce & Kirkpatrick, 2009 [23]	previous meta-analyses and reviews	no REA/GBS/IBS/IBD
Saps et al., 2008 [24]	previous meta-analyses and reviews	outcome of interest not reported
Schwille-Kiuntke et al., 2011 [25]	previous meta-analyses and reviews	outcome of interest not reported
Silva et al., 2018 [26]	databases	no REA/GBS/IBS/IBD
Söderlin et al., 2003 [27]	previous meta-analyses and reviews	outcome of interest not reported
Tuompo et al., 2020 [28]	databases	outcome of interest not reported
Uotila et al., 2011 [29]	previous meta-analyses and reviews	outcome of interest not reported
Uotila et al., 2014 [30]	previous meta-analyses and reviews	no REA/GBS/IBS/IBD
Wilson et al., 2021 [31]	databases	no REA/GBS/IBS/IBD
Wouters et al., 2016 [32]	previous meta-analyses and reviews	no results for *Campylobacter* enteritis

Calculation of the adequate sample size

The adequate sample size was calculated using the formula proposed by Daniel et al. [33]:

$$n = \frac{Z^2 P(1-P)}{d^2},$$

where *n* is the sample size, *Z* is the Z statistic for a level of confidence (i.e. 1.96 for 95 % CI), *P* is the expected proportion and *d* is the precision.

The expected proportion *P* was 1.71 % for REA, 0.07 % for GBS and 4.01 % for IBS according to the most recent pooled estimates [3,4]. For IBD the mean of the reported range of 0.05-0.45 % was used [3].

The precision *d* was set to the half of the expected proportion *P*, as *P* was below 10 % for all sequelae [34].

Accordingly, the sample size was considered to be adequate if the following CE cases were included:

- REA: ≥880 CE cases
- GBS: ≥22.000 CE cases
- CD/UC: ≥6.100 CE cases
- IBS: ≥370 CE cases.

If studies included less than 10 % of the calculated CE cases, the sample size was considered to be inadequate.

Table 3: Results of subgroup meta-analyses for the proportion of REA, GBS and IBS following *Campylobacter* infections by study size and design, sequelae diagnosis, follow-up period and *Campylobacter* species

	REA					GBS					IBS				
	**Pooled estimate**	**95% CI**	**I²**	**Number of reports**	**p-value**	**Pooled estimate**	**95% CI**	**I²**	**Number of reports**	**p-value**	**Pooled estimate**	**95% CI**	**I²**	**Number of reports**	**p-value**
**Study size**															
≤1,000 CE cases	3.61	2.15-6.00	82.5%	20	0.005	0.305	0.127-0.730	12.5%	5	0.003	9.10	5.26-15.30	86.1%	11	0.036
>1,000 CE cases	0.29	0.05-1.58	99.6%	6		0.048	0.020-0.114	95.3%	9		1.46	0.27-7.42	99.7%	6	
**Study design**															
Prospective study	4.17	2.68-6.44	94.2%	11	0.066[†]	0.265	0.100-0.705	0.0%	3	0.022	9.48	4.64-18.39	80.1%	8	0.059
Retrospective study	0.86	0.24-3.01	98.5%	14		0.055	0.022-0.138	94.4%	11		2.39	0.67-8.21	99.6%	9	
**Sequelae diagnosis**															
Confirmed by specialists/ health professionals	0.93	0.81-1.07	97.9%	17	<0.001	0.054	0.023-0.128	94.2%	11	-	1.15	0.27-4.76	99.3%	6	0.004
Self-reported	5.49	2.86-10.29	88.4%	9		-	-	-	2		10.39	5.95-17.53	85.8%	11	
**Follow-up period[‡]**															
<3 months	4.53	2.38-8.46	95.1%	10	0.002	0.189	0.046-0.778	88.9%	4	<0.001				0	0.133[†]
3 months to <1 year	2.58	0.73-8.74	89.8%	6		0.023	0.015-0.035	0.0%	3		10.17	5.08-19.33	89.5%	8	
≥1 year	0.17	0.04-0.78	98.6%	5		0.043	0.028-0.067	0.0%	4		2.21	0.56-8.26	99.0%	8	
Not reported	3.53	1.47-8.20	69.7%	5		0.368	0.138-0.977	53.4%	3		-	-	-	1	
***Campylobacter* species[§]**															
C. jejuni	3.99	2.56-6.17	79.1%	14	0.116	0.091	0.012-0.709	85.4%	6	0.945	14.55	9.07-22.52	76.3	4	0.187
Mix[¶] / undetermined	0.70	0.18-2.66	99.1%	13		0.062	0.026-0.150	95.2%	8		4.58	1.74-11.53	99.0	15	

Subgroups were only analyzed if at least three studies were included for each group.

[†] One report with missing information was excluded from analysis.

[‡] Period between CE and the onset or diagnoses of sequelae.

[§] Species-specific outcomes were added.

[¶] Mix of different *C.* species, including combinations of *C. jejuni/C. coli*, of *C. concisus/C. curvus/C. fetus/C. gracilis/C. hyointestinalis/C. lari/C. upsaliensis*, *C. spp*. in general and undetermined *C.* species.

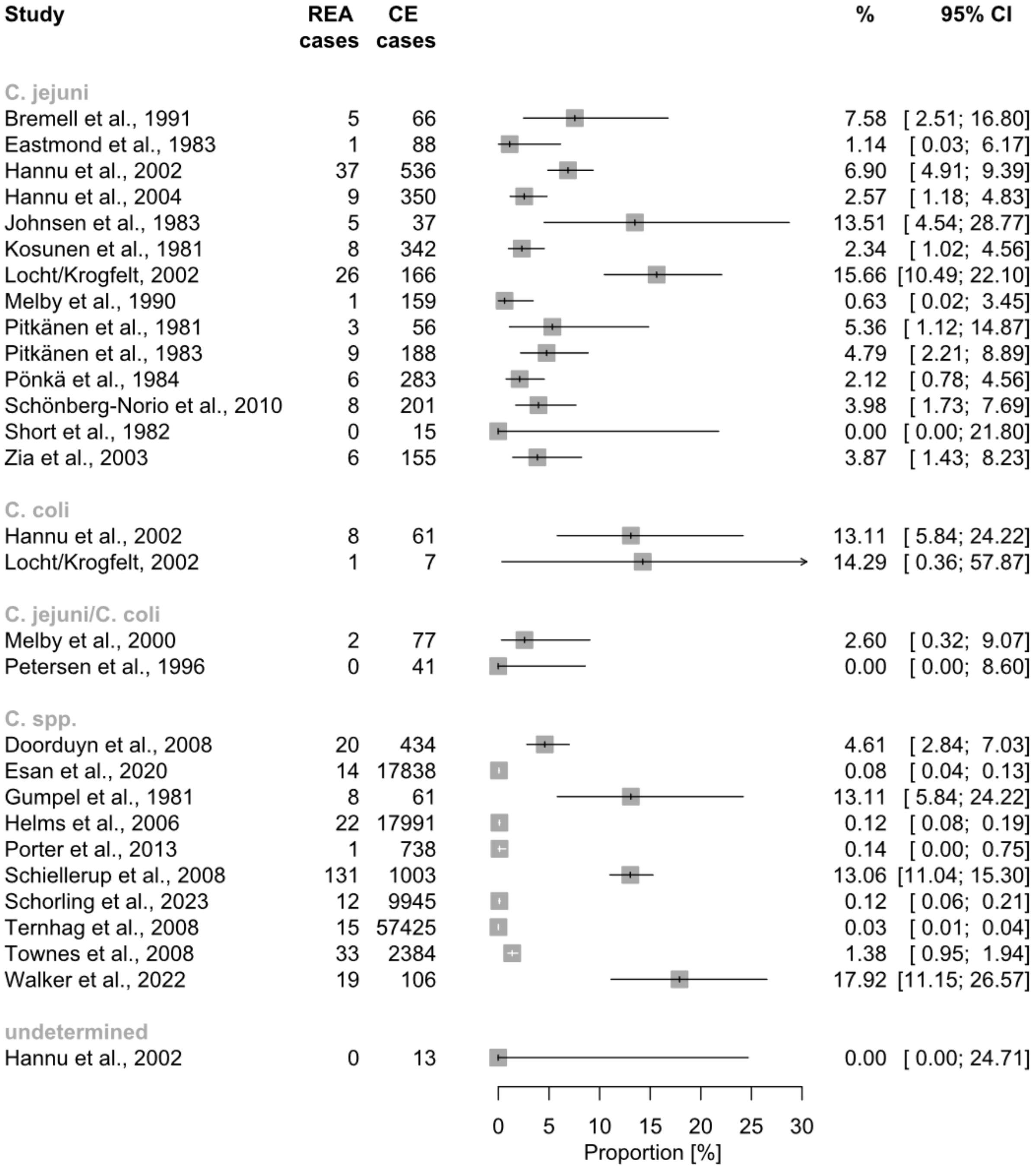

Figure 1: Forest plot of studies reporting the proportion of *Campylobacter* enteritis (CE) cases that developed reactive arthritis (REA), stratified by *C.* species

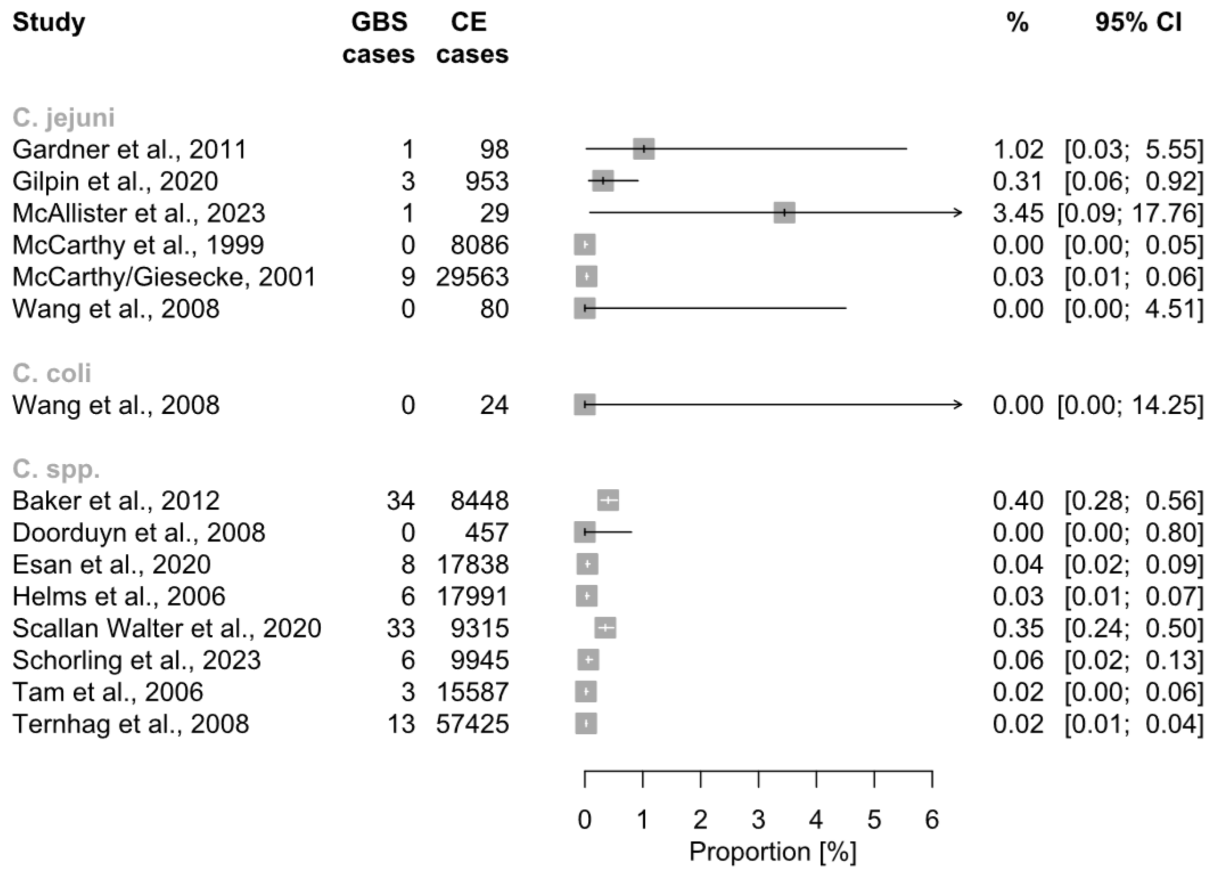

Figure 2: Forest plot of studies reporting the proportion of *Campylobacter* enteritis (CE) cases that developed Guillain-Barré syndrome (GBS), stratified by *C.* species

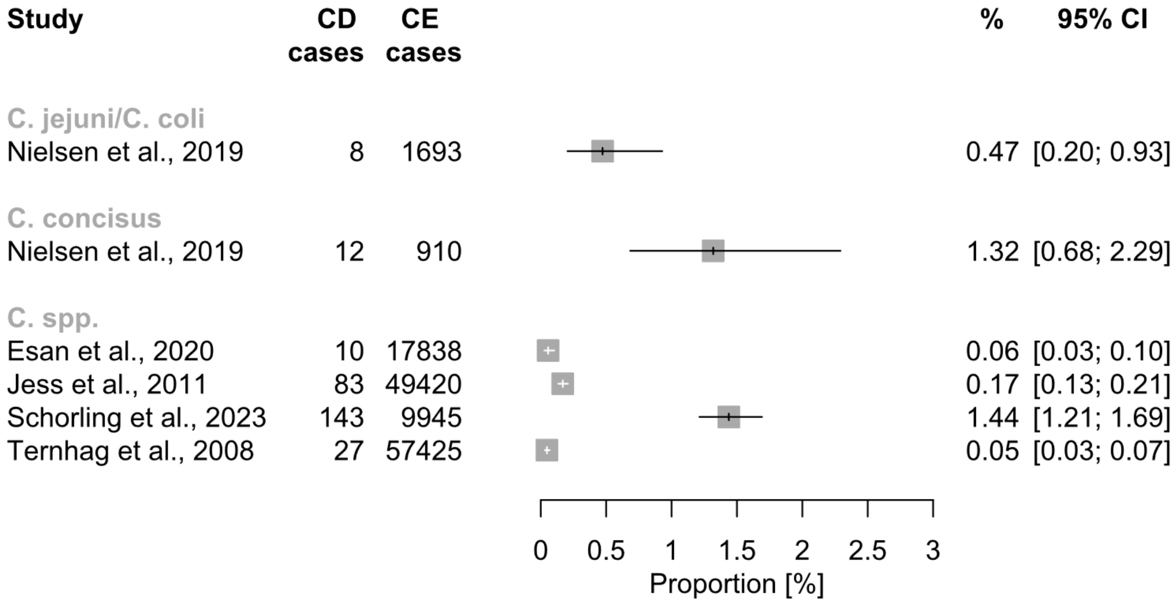

Figure 3: Forest plot of studies reporting the proportion of *Campylobacter* enteritis (CE) cases that developed Crohn's disease (CD), stratified by *C.* species

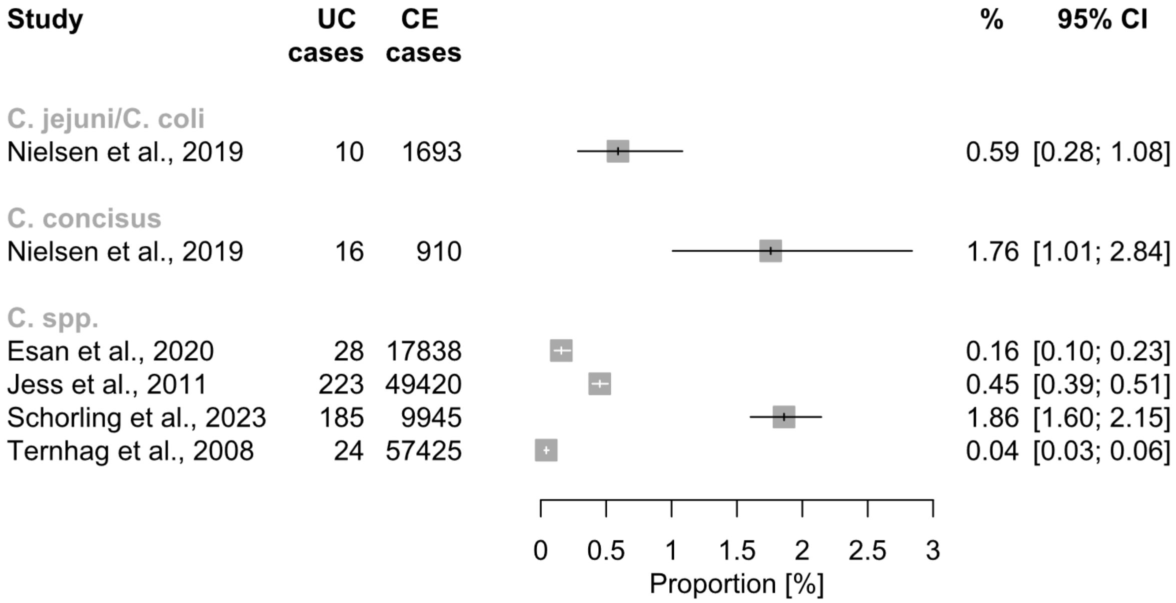

Figure 4: Forest plot of studies reporting the proportion of *Campylobacter* enteritis (CE) cases that developed ulcerative colitis (UC), stratified by *C.* species

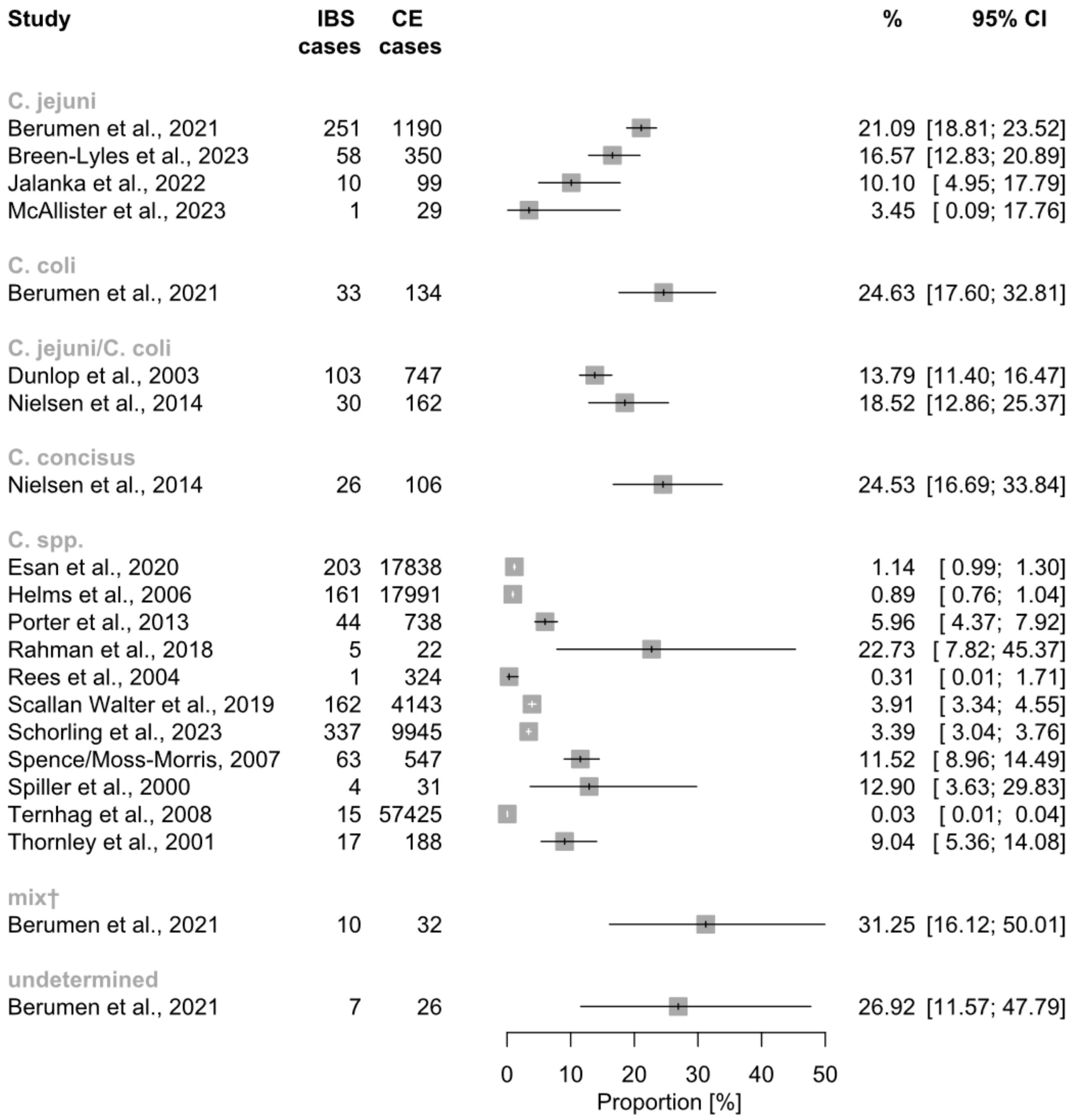

Figure 5: Forest plot of studies reporting the proportion of *Campylobacter* enteritis (CE) cases that developed irritable bowel syndrome (IBS), stratified by *C.* species

† Mix of *C. concisus, C. curvus, C. fetus, C. gracilis, C. hyointestinalis, C. lari* and *C. upsaliensis*.

Table 4: Critical appraisal of included studies

Author, year	Q1	Q2	Q3	Q4	Q5	Q6a	Q6b	Q7a	Q7b	Q8	Q9	Total 'yes'
Baker et al., 2012 [35]	yes	yes	unclear	yes	yes	yes	yes	yes	yes†	yes	not applicable	90%
Berumen et al., 2021 [36]	yes	yes	yes	yes	unclear	yes	yes	yes	yes	yes	yes	91%
Breen-Lyles et al., 2023 [37]	yes	yes	unclear	yes	unclear	yes	yes	yes	yes	yes	no	73%
Bremell et al., 1991 [38]	yes	yes	unclear	yes	unclear†	yes	no	yes	no	yes	yes	64%
Doorduyn et al., 2008 [39]	unclear	yes	no	no	no	yes	no	yes	no	yes	no	36%
Dunlop et al., 2003 [40]	yes†	yes	yes	no	unclear†	yes	yes	yes	yes	yes	no†	73%
Eastmond et al., 1983 [41]	yes	yes	unclear	no†	yes	yes	yes	yes	yes	yes	not applicable	80%
Esan et al., 2020 [42]	yes	yes	unclear	yes	yes	yes	yes	yes	yes	yes	not applicable	90%
Gardner et al., 2011 [43]	yes	yes	no	yes	yes	yes	yes	yes	yes	yes	unclear	82%
Gilpin et al., 2020 [44]	yes	yes	no	yes	yes	yes	unclear	yes	unclear	yes	not applicable	70%
Gumpel et al., 1981 [45]	yes	yes	no†	no†	yes	yes	yes	yes	yes	no	not applicable	70%
Hannu et al., 2002 [46]	yes	yes	unclear	yes	yes	yes	yes	yes	yes†	yes	yes	91%
Hannu et al., 2004 [47]	yes	yes	unclear	No	unclear	yes	yes	no	yes	Yes	unclear	55%
Helms et al., 2006 [48]	yes	yes	unclear	yes	yes	yes	yes	yes	yes	yes	not applicable	90%
Jalanka et al., 2023 [49]	yes	yes	unclear	yes	unclear	yes	yes	yes	yes	yes	no	73%
Jess et al., 2011 [50]	yes	yes	yes	yes	yes	yes	yes	yes	yes	yes	not applicable	100%
Johnsen et al., 1983 [51]	unclear	yes	no	yes	unclear	yes	yes	yes	yes	yes	yes	73%
Kosunen et al., 1981 [52]	yes	yes	unclear	no	yes	yes	yes	yes	yes	no	not applicable	70%
Locht & Krogfelt, 2002 [53]	yes	yes	unclear	yes	yes	yes	no	yes	no	yes	yes	73%
McAllister et al., 2023 [54]	yes	yes	no	yes	yes	yes	no	no	no	yes	no	55%
McCarthy et al., 1999 [55]	yes	yes	unclear	no	unclear†	yes†	yes	no	yes	yes	unclear	55%
McCarthy & Giesecke, 2001 [56]	yes	yes	yes†	no	yes	yes	yes	yes	yes	yes	not applicable	90%
Melby et al., 1990 [57]	yes	yes	unclear	yes	yes	yes	no	no	no	yes	no	55%
Melby et al., 2000 [58]	yes	yes	no	no	unclear	yes	unclear	no	unclear	yes	no	36%
Nielsen et al., 2014 [59]	yes	yes	unclear	yes	yes	yes	no	yes	no	yes	yes	73%
Nielsen et al., 2019 [60]	yes	yes	unclear	yes	yes	yes	yes	yes	yes	yes	not applicable	90%
Petersen et al., 1996 [61]	yes	yes	no†	yes	yes	yes	yes†	yes	yes	yes	not applicable	90%
Pitkänen et al., 1981 [62]	yes	yes	no	yes	yes†	yes	yes	yes	yes†	no	not applicable†	80%
Pitkänen et al., 1983 [63]	yes	yes	unclear	yes	yes	yes	yes	yes	yes	yes	not applicable†	90%

Author, year	Q1	Q2	Q3	Q4	Q5	Q6a	Q6b	Q7a	Q7b	Q8	Q9	Total 'yes'
Pönkä et al., 1984 [64]	yes	yes	unclear†	yes	yes	yes	no	yes	no	yes	yes	73%
Porter et al., 2013 [65]	no	yes	yes	yes†	yes	yes	yes	yes	yes	yes	not applicable	90%
Porter et al., 2013 [66]	no	yes	yes	no	yes	yes	yes	yes	yes	yes	not applicable	80%
Rahman et al., 2018 [67]	yes	yes	no	no	unclear	yes	yes	yes	yes	yes	no	64%
Rees et al., 2004 [68]	yes	yes	unclear	no	unclear	yes	no	yes	no	yes	yes	55%
Scallan Walter et al., 2019 [69]	yes	yes	yes	yes	yes	yes	yes	yes	yes	yes	not applicable	100%
Scallan Walter et al., 2020 [70]	yes	yes	unclear	yes	yes	yes	yes	yes	yes	yes	not applicable	90%
Schiellerup et al., 2008 [71]	yes†	yes	yes†	yes	yes	yes	yes	yes	no	yes	yes	91%
Schönberg-Norio et al., 2010 [72]	yes	yes	unclear†	yes	yes†	yes	yes	yes	yes†	yes	yes†	91%
Schorling et al., 2023 [73]	yes	yes	unclear	yes	yes	yes	yes	yes	yes	yes	not applicable	90%
Short et al., 1982 [74]	yes†	yes	no	no	yes	yes	yes	yes	yes	no	yes	73%
Spence & Moss-Morris, 2007 [75]‡	yes	yes	yes	no†	unclear†	yes	yes	yes	yes	yes	no	73%
Spiller et al., 2000 [76]	unclear†	unclear†	no	yes	yes	yes	yes	yes	yes	yes	yes	73%
Tam et al., 2006 [77]	yes	yes	unclear	no	yes	yes	yes	yes	yes	yes	not applicable	80%
Ternhag et al., 2008 [78]	yes	yes	yes	yes	yes	yes	yes	yes	yes†	yes	not applicable	100%
Thornley et al., 2001 [79]	yes	yes†	unclear	no†	unclear†	yes	yes	yes	yes	yes	yes	73%
Townes et al., 2008 [80]	yes	yes	yes†	yes	unclear†	yes	yes	yes	yes†	yes	yes	91%
Walker et al., 2022 [81]	yes	yes	unclear	yes	yes	yes	yes	yes	yes	yes	no	82%
Wang et al., 2008 [82]	yes	yes†	no	yes	yes	yes	yes	yes	yes	yes	not applicable	90%
Zia et al., 2003 [83]	yes	yes	unclear	yes	unclear	yes	no	yes	no	yes	no	55%
**Total "yes"**	90%	98%	22%	67%	67%	100%	78%	90%	76%	92%	50%§	**76%**
Reports of REA	88%	100%	15%	69%	73%	100%	73%	88%	69%	85%	60%§	75%
Reports of GBS	93%	100%	14%	71%	86%	100%	79%	86%	79%	100%	0%§	78%
Reports of CD	100%	100%	40%	100%	100%	100%	100%	100%	100%	100%	-§	94%
Reports of UC	100%	100%	40%	100%	100%	100%	100%	100%	100%	100%	-§	94%
Reports of IBS	88%	94%	35%	71%	53%	100%	82%	94%	82%	100%	45%§	78%

Critical appraisal was performed using the JBI critical appraisal tool for studies reporting prevalence data [84]. Q1: Was the sample frame appropriate to address the target population? Q2: Were study participants sampled in an appropriate way? Q3: Was the sample size adequate? Q4: Were the study subjects and the setting described in detail? Q5: Was the data analysis conducted with sufficient coverage of the identified sample? Q6: Were valid methods used for the identification of a) *Campylobacter*, b) the sequela? Q7: Was a) *Campylobacter*, b) the sequela measured in a standard, reliable way for all participants? Q8: Was there appropriate statistical analysis? Q9: Was the response rate adequate, and if not, was the low response rate managed appropriately?

† Differing appraisal compared to the evaluation performed by Esan et al. [2].

‡ The report by Moss-Morris & Spence [85] was not evaluated separately.

§ The proportion refers to reports for which the criterion was applicable.

References

1. Ajene AN, Fischer Walker CL, Black RE. Enteric pathogens and reactive arthritis: a systematic review of Campylobacter, salmonella and Shigella-associated reactive arthritis. *J Health Popul Nutr.* 2013; 31:299–307.
2. Esan OB, Pearce M, van Hecke O, Roberts N, Collins DRJ, Violato M, et al. Factors Associated with Sequelae of Campylobacter and Non-typhoidal Salmonella Infections: A Systematic Review. *EBioMedicine.* 2017; 15:100–11.
3. Keithlin J, Sargeant J, Thomas MK, Fazil A. Systematic review and meta-analysis of the proportion of Campylobacter cases that develop chronic sequelae. *BMC Public Health.* 2014; 14:1203.
4. Pogreba-Brown K, Austhof E, Tang X, Trejo MJ, Owusu-Dommey A, Boyd K, et al. Enteric Pathogens and Reactive Arthritis: Systematic Review and Meta-Analyses of Pathogen-Associated Reactive Arthritis. *Foodborne Pathog Dis.* 2021; 18:627–39.
5. Svendsen AT, Bytzer P, Engsbro AL. Systematic review with meta-analyses: does the pathogen matter in post-infectious irritable bowel syndrome. *Scand J Gastroenterol.* 2019; 54:546–62.
6. Barrett E, Carr D, Bell ML, Pogreba-Brown K. Post-infectious sequelae after Campylobacter enteric infection: a pilot study in Maricopa County, Arizona, USA. *Pilot Feasibility Stud.* 2018; 4:142.
7. Borgaonkar MR, Ford DC, Marshall JK, Churchill E, Collins SM. The incidence of irritable bowel syndrome among community subjects with previous acute enteric infection. *Dig Dis Sci.* 2006; 51:1026–32.
8. Emberland KE, Wensaas K-A, Litleskare S, Iversen A, Hanevik K, Langeland N, et al. Clinical features of gastroenteritis during a large waterborne Campylobacter outbreak in Askøy, Norway. *Infection.* 2022; 50:343–54.
9. Friesema IH, Slegers-Fitz-James IA, Wit B, Franz E. Surveillance and characteristics of food-borne outbreaks in the Netherlands, 2006 to 2019. *Euro Surveill.* 2022; 27:1–10.
10. Garg AX, Marshall J, Salvadori M, Thiessen-Philbrook HR, Macnab J, Suri RS, et al. A gradient of acute gastroenteritis was characterized, to assess risk of long-term health sequelae after drinking bacterial-contaminated water. *J Clin Epidemiol.* 2006; 59:421–8.
11. Gradel KO, Nielsen HL, Schønheyder HC, Ejlertsen T, Kristensen B, Nielsen H. Increased short- and long-term risk of inflammatory bowel disease after salmonella or campylobacter gastroenteritis. *Gastroenterology.* 2009; 137:495–501.
12. Hossain MI, Nasrin S, Das R, Palit P, Sultana A-A, Sobi RA, et al. Symptomatic and Asymptomatic Campylobacter Infections and Child Growth in South Asia: Analyzing Data from the Global Enteric Multicenter Study. *Am J Trop Med Hyg.* 2023.
13. Iacob T, Țățulescu DF, Lupșe MS, Dumitrașcu DL. Post-infectious irritable bowel syndrome after a laboratory-proven enteritis. *Exp Ther Med.* 2020; 20:3517–22.
14. Laine J, Lumio J, Toikkanen S, Virtanen MJ, Uotila T, Korpela M, et al. The duration of gastrointestinal and joint symptoms after a large waterborne outbreak of gastroenteritis in Finland in 2007--a questionnaire-based 15-month follow-up study. *PLoS One.* 2014; 9:e85457.
15. Lee H, Heo N, Kwon D, Ha J. Deciphering changes in the incidence of the Guillain-Barré syndrome during the COVID-19 pandemic: a nationwide time-series correlation study. *BMJ Neurol Open.* 2022; 4:e000378.
16. Li Y, Zhou G, Gao P, Gu Y, Wang H, Zhang S, et al. Gastroenteritis Outbreak Caused by Campylobacter jejuni - Beijing, China, August, 2019. *China CDC Wkly.* 2020; 2:422–5.
17. Marshall JK, Thabane M, Garg AX, Clark WF, Moayyedi P, Collins SM. Eight year prognosis of postinfectious irritable bowel syndrome following waterborne bacterial dysentery. *Gut.* 2010; 59:605–11.
18. Neal KR, Hebden J, Spiller R. Prevalence of gastrointestinal symptoms six months after bacterial gastroenteritis and risk factors for development of the irritable bowel syndrome: postal survey of patients. *BMJ.* 1997; 314:779–82.
19. Neal KR, Barker L, Spiller RC. Prognosis in post-infective irritable bowel syndrome: a six year follow up study. *Gut.* 2002; 51:410–3.
20. O'Connor L, McKeown P, Barrasa A, Garvey P. Epidemiology of Campylobacter infections in Ireland 2004-2016: What has changed. *Zoonoses Public Health.* 2020; 67:362–9.
21. Omarova S, Awad K, Moos V, Püning C, Gölz G, Schulzke J-D, et al. Intestinal Barrier in Post-Campylobacter jejuni Irritable Bowel Syndrome. *Biomolecules.* 2023; 13.
22. Parry SD, Stansfield R, Jelley D, Gregory W, Phillips E, Barton, JR, et al. Does bacterial gastroenteritis predispose people to functional gastrointestinal disorders? A prospective, community-based, case-control study. *Am J Gastroenterol.* 2003; 98:1970–5.
23. Pierce KK, Kirkpatrick BD. Update on human infections caused by intestinal protozoa. *Curr Opin Gastroenterol.* 2009; 25:12–7.
24. Saps M, Pensabene L, Di Martino L, Staiano A, Wechsler J, Zheng X, et al. Post-infectious functional gastrointestinal disorders in children. *J Pediatr.* 2008; 152:812-6, 816.e1.

25. Schwille-Kiuntke J, Enck P, Zendler C, Krieg M, Polster AV, Klosterhalfen S, et al. Postinfectious irritable bowel syndrome: follow-up of a patient cohort of confirmed cases of bacterial infection with Salmonella or Campylobacter. *Neurogastroenterol Motil.* 2011; 23:e479-88.
26. Silva WC, Targino BN, Mendonca RS, Sant'Ana AS, Hungaro HM. Campylobacter: An overview of cases, occurrence in food, contamination sources, and antimicrobial resistance in Brazil. *FOOD REVIEWS INTERNATIONAL.* 2018; 34:364–89.
27. Söderlin MK, Kautiainen H, Jonsson D, Skogh T, Leirisalo-Repo M. The costs of early inflammatory joint disease: a population-based study in southern Sweden. *Scand J Rheumatol.* 2003; 32:216–24.
28. Tuompo R, Lääveri T, Hannu T, Pakkanen SH, Kirveskari J, Leirisalo-Repo M, et al. Reactive arthritis and other musculoskeletal symptoms associated with acquisition of diarrhoeagenic Escherichia coli (DEC). *Ann Rheum Dis.* 2020; 79:605–11.
29. Uotila T, Antonen J, Laine J, Kujansuu E, Haapala A-M, Lumio J, et al. Reactive arthritis in a population exposed to an extensive waterborne gastroenteritis outbreak after sewage contamination in Pirkanmaa, Finland. *Scand J Rheumatol.* 2011; 40:358–62.
30. Uotila T, Korpela M, Vuento R, Laine J, Lumio J, Kuusi M, et al. Joint symptoms after a faecal culture positive Campylobacter infection associated with a waterborne gastroenteritis outbreak: a questionnaire study. *Scand J Rheumatol.* 2014; 43:524–6.
31. Wilson B, Jones N, Wood T, Jagroop-Dearing A, Kubovy J, Baker MG. Clinical outcomes of campylobacteriosis: a case series analysis of hospitalisations associated with the Havelock North Campylobacter outbreak. *N Z Med J.* 2021; 134:71–84.
32. Wouters MM, van Wanrooy S, Nguyen A, Dooley J, Aguilera-Lizarraga J, van Brabant W, et al. Psychological comorbidity increases the risk for postinfectious IBS partly by enhanced susceptibility to develop infectious gastroenteritis. *Gut.* 2016; 65:1279–88.
33. Daniel WW. Biostatistics: A Foundation for Analysis in the Health Sciences. 7th ed. New York: John Wiley & Sons; 1999.
34. Naing L, Winn T, Rusli BN. Practical issues in calculating the sample size for prevalence studies. *Arch Orofac Sci.* 2006:9-14.
35. Baker MG, Kvalsvig A, Zhang J, Lake R, Sears A, Wilson N. Declining Guillain-Barré syndrome after campylobacteriosis control, New Zealand, 1988-2010. *Emerg Infect Dis.* 2012; 18:226–33.
36. Berumen A, Lennon R, Breen-Lyles M, Griffith J, Patel R, Boxrud D, et al. Characteristics and Risk Factors of Post-Infection Irritable Bowel Syndrome After Campylobacter Enteritis. *Clin Gastroenterol Hepatol.* 2021; 19:1855-1863.e1.
37. Breen-Lyles M, Decuir M, Byale A, Smith K, Grover M. Impact of Rome IV criteria on the prevalence of post-infection irritable bowel syndrome. *Neurogastroenterol Motil.* 2023:e14532.
38. Bremell T, Bjelle A, Svedhem A. Rheumatic symptoms following an outbreak of campylobacter enteritis: a five year follow up. *Ann Rheum Dis.* 1991; 50:934–8.
39. Doorduyn Y, van Pelt W, Siezen CLE, van der Horst F, Van Duynhoven, Y T H P, Hoebee B, et al. Novel insight in the association between salmonellosis or campylobacteriosis and chronic illness, and the role of host genetics in susceptibility to these diseases. *Epidemiol Infect.* 2008; 136:1225–34.
40. Dunlop SP, Jenkins D, Neal KR, Spiller RC. Relative importance of enterochromaffin cell hyperplasia, anxiety, and depression in postinfectious IBS. *Gastroenterology.* 2003; 125:1651–9.
41. Eastmond CJ, Rennie JA, Reid TM. An outbreak of Campylobacter enteritis--a rheumatological followup survey. *J Rheumatol.* 1983; 10:107–8.
42. Esan OB, Perera R, McCarthy N, Violato M, Fanshawe TR. Incidence, risk factors, and health service burden of sequelae of campylobacter and non-typhoidal salmonella infections in England, 2000-2015: A retrospective cohort study using linked electronic health records. *J Infect.* 2020; 81:221–30.
43. Gardner TJ, Fitzgerald C, Xavier C, Klein R, Pruckler J, Stroika S, et al. Outbreak of campylobacteriosis associated with consumption of raw peas. *Clin Infect Dis.* 2011; 53:26–32.
44. Gilpin BJ, Walker T, Paine S, Sherwood J, Mackereth G, Wood T, et al. A large scale waterborne Campylobacteriosis outbreak, Havelock North, New Zealand. *J Infect.* 2020; 81:390–5.
45. Gumpel JM, Martin C, Sanderson PJ. Reactive arthritis associated with campylobacter enteritis. *Ann Rheum Dis.* 1981; 40:64–5.
46. Hannu T, Mattila L, Rautelin H, Pelkonen P, Lahdenne P, Siitonen A, et al. Campylobacter-triggered reactive arthritis: a population-based study. *Rheumatology (Oxford).* 2002; 41:312–8.
47. Hannu T, Kauppi M, Tuomala M, Laaksonen I, Klemets P, Kuusi M. Reactive arthritis following an outbreak of Campylobacter jejuni infection. *J Rheumatol.* 2004; 31:528–30.
48. Helms M, Simonsen J, Mølbak K. Foodborne bacterial infection and hospitalization: a registry-based study. *Clin Infect Dis.* 2006; 42:498–506.

49. Jalanka J, Gunn D, Singh G, Krishnasamy S, Lingaya M, Crispie F, et al. Postinfective bowel dysfunction following Campylobacter enteritis is characterised by reduced microbiota diversity and impaired microbiota recovery. *Gut.* 2023; 72:451–9.
50. Jess T, Simonsen J, Nielsen NM, Jørgensen KT, Bager P, Ethelberg S, et al. Enteric Salmonella or Campylobacter infections and the risk of inflammatory bowel disease. *Gut.* 2011; 60:318–24.
51. Johnsen K, Ostensen M, Melbye AC, Melby K. HLA-B27-negative arthritis related to Campylobacter jejuni enteritis in three children and two adults. *Acta Med Scand.* 1983; 214:165–8.
52. Kosunen TU, Pönkä A, Kauranen O, Martio J, Pitkänen T, Hortling L, et al. Arthritis associated with Campylobacter jejuni enteritis. *Scand J Rheumatol.* 1981; 10:77–80.
53. Locht H, Krogfelt KA. Comparison of rheumatological and gastrointestinal symptoms after infection with Campylobacter jejuni/coli and enterotoxigenic Escherichia coli. *Ann Rheum Dis.* 2002; 61:448–52.
54. McAllister J, Gregory J, Adamopoulos J, Walsh M, Stylianopoulos A, Arnold A-L, et al. A foodborne outbreak of campylobacteriosis at a wedding - Melbourne, Australia, 2022. *Communicable diseases intelligence.* 2023; 47.
55. McCarthy N, Andersson Y, Jormanainen V, Gustavsson O, Giesecke J. The risk of Guillain-Barré syndrome following infection with Campylobacter jejuni. *Epidemiol Infect.* 1999; 122:15–7.
56. McCarthy N, Giesecke J. Incidence of Guillain-Barré syndrome following infection with Campylobacter jejuni. *Am J Epidemiol.* 2001; 153:610–4.
57. Melby K, Dahl OP, Crisp L, Penner JL. Clinical and serological manifestations in patients during a waterborne epidemic due to Campylobacter jejuni. *J Infect.* 1990; 21:309–16.
58. Melby KK, Svendby JG, Eggebø T, Holmen LA, Andersen BM, Lind L, et al. Outbreak of Campylobacter infection in a subartic community. *Eur J Clin Microbiol Infect Dis.* 2000; 19:542–4.
59. Nielsen HL, Engberg J, Ejlertsen T, Nielsen H. Psychometric scores and persistence of irritable bowel after Campylobacter concisus infection. *Scand J Gastroenterol.* 2014; 49:545–51.
60. Nielsen HL, Dalager-Pedersen M, Nielsen H. Risk of inflammatory bowel disease after Campylobacter jejuni and Campylobacter concisus infection: a population-based cohort study. *Scand J Gastroenterol.* 2019; 54:265–72.
61. Petersen AM, Nielsen SV, Meyer D, Ganer P, Ladefoged K. Bacterial gastroenteritis among hospitalized patients in a Danish County, 1991-93. *Scand J Gastroenterol.* 1996; 31:906–11.
62. Pitkänen T, Pettersson T, Pönkä A, Kosunen TU. Clinical and serological studies in patients with Campylobacter fetus ssp. jejuni infection: I. Clinical findings. *Infection.* 1981; 9:274–8.
63. Pitkänen T, Pönkä A, Pettersson T, Kosunen TU. Campylobacter enteritis in 188 hospitalized patients. *Arch Intern Med.* 1983; 143:215–9.
64. Pönkä A, Pitkänen T, Sarna S, Kosunen TU. Infection due to Campylobacter jejuni: a report of 524 outpatients. *Infection.* 1984; 12:175–8.
65. Porter CK, Choi D, Riddle MS. Pathogen-specific risk of reactive arthritis from bacterial causes of foodborne illness. *J Rheumatol.* 2013; 40:712–4.
66. Porter CK, Choi D, Cash B, Pimentel M, Murray J, May L, et al. Pathogen-specific risk of chronic gastrointestinal disorders following bacterial causes of foodborne illness. *BMC Gastroenterol.* 2013; 13:46.
67. Rahman MM, Ghoshal UC, Sultana S, Kibria MG, Sultana N, Khan ZA, et al. Long-Term Gastrointestinal Consequences are Frequent Following Sporadic Acute Infectious Diarrhea in a Tropical Country: A Prospective Cohort Study. *Am J Gastroenterol.* 2018; 113:1363–75.
68. Rees JR, Pannier MA, McNees A, Shallow S, Angulo FJ, Vugia DJ. Persistent diarrhea, arthritis, and other complications of enteric infections: a pilot survey based on California FoodNet surveillance, 1998-1999. *Clin Infect Dis.* 2004; 38 Suppl 3:S311-7.
69. Scallan Walter EJ, Crim SM, Bruce BB, Griffin PM. Postinfectious Irritable Bowel Syndrome After Campylobacter Infection. *American Journal of Gastroenterology.* 2019; 114:1649–56.
70. Scallan Walter EJ, Crim SM, Bruce BB, Griffin PM. Incidence of Campylobacter-Associated Guillain-Barre Syndrome Estimated from Health Insurance Data. *Foodborne Pathog Dis.* 2020; 17:23–8.
71. Schiellerup P, Krogfelt KA, Locht H. A comparison of self-reported joint symptoms following infection with different enteric pathogens: effect of HLA-B27. *J Rheumatol.* 2008; 35:480–7.
72. Schönberg-Norio D, Mattila L, Lauhio A, Katila M-L, Kaukoranta S-S, Koskela M, et al. Patient-reported complications associated with Campylobacter jejuni infection. *Epidemiol Infect.* 2010; 138:1004–11.
73. Schorling E, Lick S, Steinberg P, Brüggemann DA. Health care utilizations and costs of Campylobacter enteritis in Germany: A claims data analysis. *PLoS One.* 2023; 18:e0283865.
74. Short CD, Klouda PT, Smith L. Campylobacter jejuni enteritis and reactive arthritis. *Ann Rheum Dis.* 1982; 41:287–8.
75. Spence MJ, Moss-Morris R. The cognitive behavioural model of irritable bowel syndrome: a prospective investigation of patients with gastroenteritis. *Gut.* 2007; 56:1066–71.

76. Spiller RC, Jenkins D, Thornley JP, Hebden JM, Wright T, Skinner M, et al. Increased rectal mucosal enteroendocrine cells, T lymphocytes, and increased gut permeability following acute Campylobacter enteritis and in post-dysenteric irritable bowel syndrome. *Gut.* 2000; 47:804–11.
77. Tam CC, Rodrigues LC, Petersen I, Islam A, Hayward A, O'Brien SJ. Incidence of Guillain-Barré syndrome among patients with Campylobacter infection: a general practice research database study. *J Infect Dis.* 2006; 194:95–7.
78. Ternhag A, Törner A, Svensson A, Ekdahl K, Giesecke J. Short- and long-term effects of bacterial gastrointestinal infections. *Emerg Infect Dis.* 2008; 14:143–8.
79. Thornley JP, Jenkins D, Neal K, Wright T, Brough J, Spiller RC. Relationship of Campylobacter toxigenicity in vitro to the development of postinfectious irritable bowel syndrome. *J Infect Dis.* 2001; 184:606–9.
80. Townes JM, Deodhar AA, Laine ES, Smith K, Krug HE, Barkhuizen A, et al. Reactive arthritis following culture-confirmed infections with bacterial enteric pathogens in Minnesota and Oregon: a population-based study. *Ann Rheum Dis.* 2008; 67:1689–96.
81. Walker TA, Grainger R, Quirke T, Roos R, Sherwood J, Mackereth G, et al. Reactive arthritis incidence in a community cohort following a large waterborne campylobacteriosis outbreak in Havelock North, New Zealand. *BMJ Open.* 2022; 12:e060173.
82. Wang S-C, Chang L-Y, Hsueh P-R, Lu C-Y, Lee P-I, Shao P-L, et al. Campylobacter enteritis in children in northern Taiwan--a 7-year experience. *J Microbiol Immunol Infect.* 2008; 41:408–13.
83. Zia S, Wareing D, Sutton C, Bolton E, Mitchell D, Goodacre JA. Health problems following Campylobacter jejuni enteritis in a Lancashire population. *Rheumatology (Oxford).* 2003; 42:1083–8.
84. Munn Z, Moola S, Lisy K, Riitano D, Tufanaru C. Methodological guidance for systematic reviews of observational epidemiological studies reporting prevalence and cumulative incidence data. *Int J Evid Based Healthc.* 2015; 13:147–53.
85. Moss-Morris R, Spence M. To "lump" or to "split" the functional somatic syndromes: can infectious and emotional risk factors differentiate between the onset of chronic fatigue syndrome and irritable bowel syndrome. *Psychosom Med.* 2006; 68:463–9.
